# Nanomaterial-mediated regulation of ion channels and calcium-centered ionic homeostasis in cancer therapy

**DOI:** 10.1186/s12951-026-04893-2

**Published:** 2026-08-12

**Authors:** Meiyan Zou, Nina Li, Xu Chen, Zhiyu Guo, Rongwei Xu, Zihao Zhou, Xinyuan Zhao, Li Cui

**Affiliations:** 1 School of Stomatology, Stomatological Hospital, Southern Medical University, Guangzhou, 510280 Guangdong China; 2 School of Dentistry, University of California, Los Angeles, Los Angeles, CA 90095 USA

**Keywords:** Ion channels, Cancer nanomedicine, Ionic homeostasis, Stimulus-responsive nanomaterials, Calcium overload

## Abstract

Ion channels govern membrane excitability, ionic gradients and intracellular signaling in cancer cells, with broad consequences for tumor progression, cell death, therapeutic resistance and remodeling of the tumor microenvironment. Yet conventional pharmacological agonists and blockers, as well as genetic interventions, often lack the spatial and temporal precision required for selective cancer therapy. Nanomaterials offer a route to overcome these limitations by confining ion channel modulation to tumor tissues, cellular membranes or subcellular organelles, and by enabling externally triggered or tumor-responsive control of channel activation, inhibition, silencing or engineered ion-transport. This review summarizes recent advances in nanomaterial-mediated regulation of ion channels and ionic homeostasis in cancer therapy, with particular attention to calcium-centered channel regulation and calcium homeostasis disruption, while also covering emerging K⁺, Na⁺, Cl^⁻^ and engineered ion-transport strategies. Across these platforms, programmable ionic perturbations can drive calcium overload, mitochondrial dysfunction, chloride imbalance, membrane potential collapse, apoptosis, immune remodeling and reversal of therapeutic resistance. These studies position nanomaterials not merely as delivery vehicles, but as active regulators of ionic circuits in cancer. Future work should define ionic dose, tumor selectivity, response durability and long-term safety with greater precision to enable the transition of ion channel nanomedicine from proof-of-concept studies to reproducible therapeutic translation.

**Graphical Abstract:**

**Figure:**
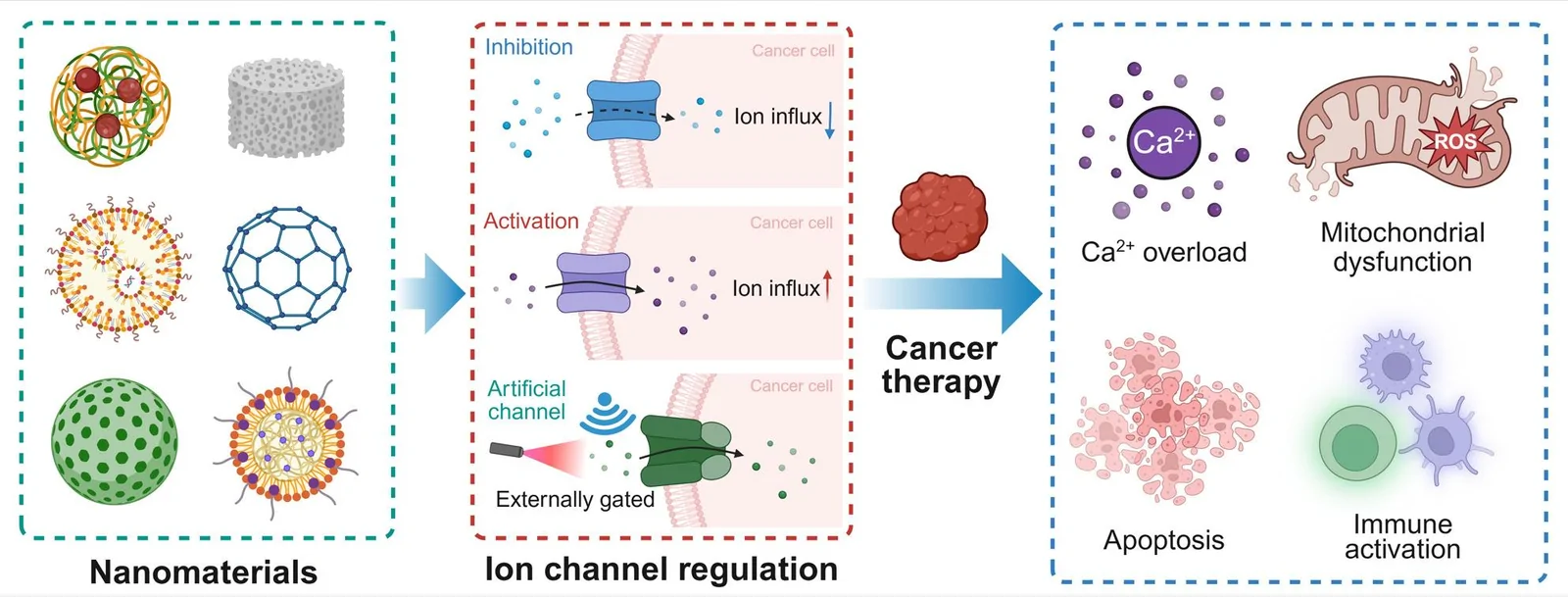

## Introduction

Ion channels are transmembrane proteins that selectively conduct ions such as K^+^, Na^+^, Ca^2+^ and Cl^⁻^ across biological membranes. By controlling ion fluxes, membrane potential (V_mem_) and electrochemical gradients, they regulate diverse cellular processes, including membrane excitability, metabolism and intracellular signaling. In cancer, ion channels are not merely passive membrane pores, but integral components of the bioelectric machinery that shapes malignant phenotypes [1]. Altered expression of ion channels and transporters has therefore emerged as a potential source of cancer biomarkers, as channel profiles can reflect tumor type, disease progression and malignant potential [2]. During tumor progression, remodeling of K^+^, Na^+^, Ca^2+^ and Cl^⁻^ channels reshapes V_mem_, ionic gradients and signaling networks [3]. These alterations influence proliferation, migration, invasion, apoptotic resistance and therapeutic response, positioning ion channels as regulatory nodes that couple ionic homeostasis to malignant phenotypes rather than as isolated membrane-protein targets [4]. Beyond individual cellular processes, ion channels also shape tumor microenvironment adaptation by linking ionic signaling to metabolism, mitochondrial function, mechanosensitive pathways and tumor–immune communication. This systems-level role highlights the therapeutic potential of controlling ionic fluxes and membrane excitability within the tumor microenvironment.

Conventional ion channel pharmacology has provided many agonists and blockers, but these agents are poorly matched to the spatial and temporal requirements of cancer therapy. Many cancer-relevant channels are also functional in healthy cells, so systemic channel modulation does not automatically restrict ion channel regulation to malignant tissue [5]. Free small-molecule modulators are mainly governed by circulation, diffusion and bulk concentration, whereas effective ion channel therapy often requires regulation at a defined membrane region, organelle or stimulation window [6]. This limitation becomes especially important for intracellular targets. Mitochondrial K_V_1.3 can be pharmacologically targeted to induce cancer cell death [7], but later nanoplatform designs combined mitochondrial potassium channel regulation with tumor accumulation, mitochondrial localization and near-infrared (NIR)-triggered release [8]. Thus, the key limitation of conventional ion channel modulators is not a lack of intrinsic pharmacological activity, but their limited ability to confine this activity to the desired tissue, subcellular compartment and therapeutic time window.

**Fig. 1:**
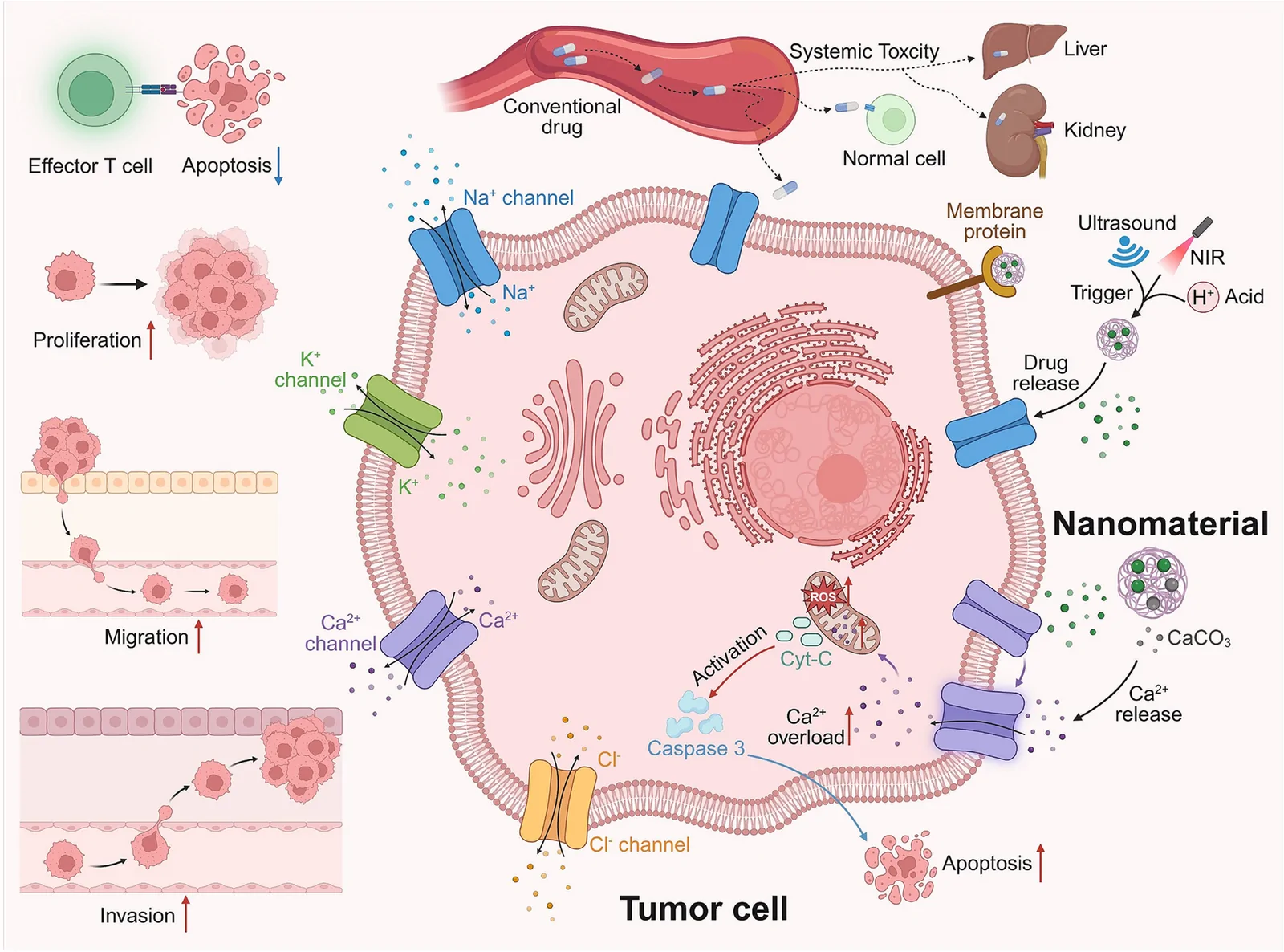
Nanomaterial-mediated regulation of ion channel signaling and ionic homeostasis in cancer therapy. Ion channels regulate ionic homeostasis, membrane potential and intracellular signaling, thereby shaping tumor cell proliferation, migration, invasion, apoptosis and interactions with the tumor microenvironment. Conventional ion channel modulators are limited by nonspecific distribution and systemic toxicity, whereas nanomaterials enable more localized and stimulus-responsive intervention. Through tumor targeting, membrane protein recognition, controlled drug release and ion supply functions, nanoplatforms can regulate Na^+^, K^+^, Ca^2+^ and Cl^⁻^ fluxes by modulating channel activity, guiding drug delivery or directly disrupting ionic homeostasis. These ion channel-centered strategies disrupt ionic balance, amplify tumor cell stress and enhance therapeutic responses, including apoptosis, inhibition of malignant progression and immune-mediated tumor killing

Nanomaterials address this limitation by converting ion channel modulation from diffuse systemic exposure into controlled local intervention. Membrane-proximal release and organelle-targeted design can place channel regulation closer to tumor cell membranes or intracellular channel sites, improving spatial selectivity over free-drug distribution [9, 10]. Responsive material design further adds temporal control, allowing light, ultrasound or optogenetic activation to trigger channel activation, blockade, drug release or engineered conductance under defined external cues [11, 12]. Nanomaterials can also couple channel regulation with local ion supply or material-generated photodynamic stress, so that channel-mediated ionic flux is amplified into calcium overload, mitochondrial dysfunction or apoptotic signaling rather than remaining a standalone pharmacological event [13, 14]. In this way, nanomaterials shift ion channel therapy from simple channel targeting toward controlled ionic intervention, where ion flux can be regulated with greater spatial and temporal precision. **(**Fig. 1**).**

Previous reviews have discussed nanoparticle-mediated ion channel manipulation [6], calcium homeostasis regulation [15], calcium-overload therapy [16] and metal ion interference therapy [17]. These studies provide important perspectives on nanoparticle–channel interactions, Ca^2+^-centered nanotherapy and metal-ion interference. The present review takes a cancer nanomedicine-oriented focus by examining how nanomaterials use ion channels and ionic homeostasis as therapeutic intervention points. Specifically, we distinguish ion channels as direct regulatory targets, channel expression as a guide for selective delivery, ionic-homeostasis disruption as a downstream stress mechanism, and engineered ion-transport systems as introduced conductance pathways. Rather than focusing on a single ion type or interference mode, we discuss K^+^/Na^+^, Ca^2+^, Cl^⁻^ and engineered ion-transport strategies according to how nanomaterials initiate, localize and translate ionic perturbations into antitumor effects. Because calcium flux serves both as an ionic event and an intracellular signal, calcium-centered nanotherapies currently represent the most extensively developed branch of this field. Accordingly, this review gives proportionally greater attention to Ca^2+^ channel regulation and calcium-overload mechanisms, while also discussing emerging K^+^, Na^+^, Cl^⁻^ and engineered ion-transport strategies according to their therapeutic approaches. Across these systems, we examine how material design controls the site, timing and downstream consequence of channel regulation, and how these controlled ionic events are translated into calcium overload, mitochondrial dysfunction, V_mem_ disruption, apoptosis or immune modulation. This framing helps clarify how channel biology and material-controlled intervention converge to generate antitumor ionic stress.

## Ion channels as determinants of cancer progression and therapy vulnerability

Ion channels influence cancer progression by shaping malignant cell states rather than acting as isolated conductance pathways. Altered Na_V_1.5 expression is associated with increased metastasis and poorer cancer-specific survival in breast cancer, suggesting that sodium channel remodeling accompanies clinically aggressive disease [18]. Beyond sodium channels, a few representative examples from potassium, calcium and mechanosensitive channels further illustrate how ion channel dysregulation can participate in distinct aspects of tumor biology and provide useful context for the nanomedicine strategies discussed below. Potassium channel dysregulation can reinforce proliferative and metastatic capacity, as *KCNK9* overexpression predicts poorer survival and *KCNK9* downregulation suppresses proliferation, migration, invasion and hepatic metastasis in colon cancer models [19]. Channel-dependent calcium entry contributes to tumor progression through stem-like programs, as ORAI3 promotes cancer stemness through ID1 upregulation in oral/oropharyngeal squamous cell carcinoma [20]. As tumor cells encounter mechanically heterogeneous tissues, Piezo type mechanosensitive ion channel component 1 (PIEZO1) activation promotes cervical cancer cell invasion and migration through the release of adenosine triphosphate (ATP) into the extracellular space [21].

Ion channels further determine whether these malignant states are maintained as survival advantages or exposed as cell-death vulnerabilities. Transmembrane member 16A (TMEM16A)/Anoctamin-1 (ANO1) supports prostate cancer cell growth, whereas its inhibition activates TNF-α-associated apoptotic signaling [22]. Sustained Ca^2+^ entry can reinforce tumor expansion when coupled to growth-promoting pathways, as shown by TRPV6-driven breast cancer migration and metastasis through NFATC2IP/NFATC2 signaling [23]. This control over cell fate extends from the plasma membrane to intracellular compartments. Mitochondrial K_V_1.3 creates an organelle-level vulnerability, because pharmacological targeting of this channel disrupts mitochondrial function and selectively kills tumor cells in vivo [7]. Thus, the same ion channel systems that help cancer cells preserve growth and adaptation can also define points at which malignant cells become susceptible to ionic imbalance, mitochondrial stress or apoptosis.

Therapeutic pressure further exposes these ionic dependencies. The transient receptor potential canonical 6 (TRPC6) channel-mediated Ca^2+^ entry supports chemotherapy-induced persistence in breast cancer by regulating integrin α6 mRNA splicing, allowing residual treatment-tolerant cells to emerge [24]. Beyond chemotherapy, K_Ca_3.1 activity confers radioresistance in breast cancer cells, indicating that channel-regulated ionic states can affect response to physical injury as well [25]. These cancer-biological roles establish ion channels as key regulators of progression and therapeutic vulnerability. Because channel effects depend on subtype, cellular location and timing, systemic modulation alone rarely provides sufficient spatial or temporal control. This limitation creates the rationale for nanomaterial-mediated channel regulation, where activity can be confined to tumor or subcellular sites and triggered under defined external stimulation.

## K^+^/Na^+^ channel regulation for nanomaterial-based cancer therapy

Malignant cells remodel K^+^ and Na^+^ conductance to sustain survival, resist apoptosis and promote dissemination [26, 27]. However, because these channels also support essential physiological functions in normal tissues [28], their therapeutic modulation requires precise spatial and temporal control. Nanomaterial-based strategies address this challenge by confining channel activation or blockade to tumor sites, silencing pro-metastatic channel expression, or exploiting aberrant channel expression for selective therapeutic delivery.

### Channel activation

**Fig. 2:**
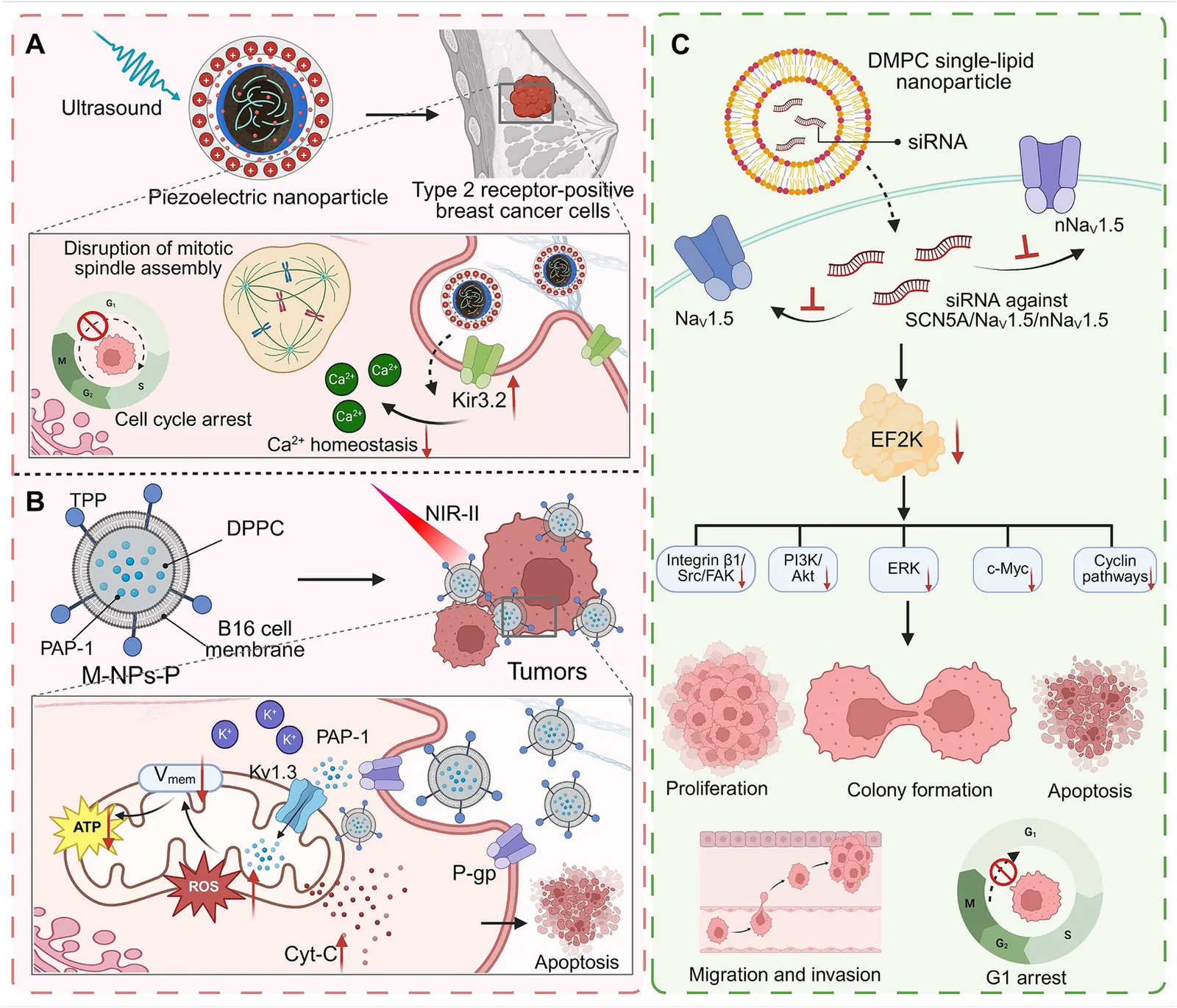
Nanomaterial-mediated regulation of K^+^/Na^+^ channels in ion channel-centered cancer therapy. (**A**) Ultrasound-activated piezoelectric nanoparticles remotely stimulate HER2-positive breast cancer cells, upregulating K_ir_3.2 and disrupting Ca^2+^ homeostasis, cell cycle progression and spindle assembly. (**B**) Biomimetic NIR-II-responsive nanoparticles target mitochondria and release PAP-1 under 1060 nm irradiation, inhibiting K_V_1.3 and inducing ROS accumulation, mitochondrial dysfunction and apoptosis. (**C**) DMPC lipid nanoparticles deliver siRNA against Na_V_1.5/nNa_V_1.5 in TNBC, suppressing EF2K-related oncogenic signaling and reducing proliferation, migration and invasion

Channel activation reprograms tumor bioelectric signaling by increasing K^+^ channel activity or channel-associated conductance responses. Chitosan/pluronic smart injectable hydrogels loaded with the K_V_11.1 activator NS1643 (SIHD-Ka) localize K^+^ channel activation in triple-negative breast cancer. The thermosensitive hydrogel forms an injectable depot and releases intact NS1643 over time under physiological conditions. Sustained NS1643 exposure pharmacologically activates K_V_11.1-dependent bioelectric regulation, arresting MDA-MB-231 cells in G0/G1 phase and limiting progression into S/G2/M phases. This localized channel activation suppresses TNBC growth, reducing tumor cell viability and tumor burden while lowering PCNA-positive proliferative activity without obvious systemic toxicity [29]. Channel activation can also be driven by physical stimulation. Ultrasound-activated anti-HER2-functionalized barium titanate piezoelectric nanoparticles (Ab-BTNPs) remotely stimulate human epidermal growth factor type 2 receptor (HER2)-positive breast cancer cells and upregulate *KCNJ6*/K_ir_3.2 inward-rectifier K^+^ channels. The resulting electrical input increases intracellular Ca^2+^ levels as part of the downstream ionic response, induces G0/G1 cell cycle arrest and disrupts mitotic spindle organization. This electrically mediated K^+^ channel-associated modulation reduces Ki-67 expression and breast cancer cell proliferation without relying on conventional cytotoxic drug release **(**Fig. 2A**)** [30].

### Localized channel blockade

Localized channel blockade is used when K^+^ channel inhibition must be restricted to the tumor region, a specific organelle or an immune niche, rather than broadly suppressing physiological excitability. Biomimetic NIR-II-responsive conjugated oligomer nanoparticles regulate mitochondrial K_V_1.3 channels through light-triggered release of PAP-1, a selective K_V_1.3 inhibitor. The melanoma cell membrane coating promotes tumor accumulation, while triphenylphosphonium (TPP) guides the nanoparticles into mitochondria. Upon 1060 nm irradiation, photothermal 1,2-dipalmitoyl-sn-glycerol-3-phosphocholine (DPPC) phase transition releases PAP-1 within mitochondria, blocking mitochondrial K^+^ transport. K_V_1.3 inhibition elevates oxygen species (ROS), collapses mitochondrial membrane potential, reduces ATP production and promotes cytochrome C (Cyt-C) release, thereby driving apoptosis and suppressing melanoma growth in vivo **(**Fig. 2B**)** [8]. Similarly, cross-linked poly-2-Hydroxyethyl methacrylate (HEMA)-pectin nanogels (HPN) deliver potassium channel blockers dofetilide and azimilide to tumor cells with controlled release and tumor-compatible size (~ 230 nm). Lysosomal accumulation and sustained release enhance selective cytotoxicity in A549 lung cancer cells while sparing normal bronchial epithelial cells. Co-delivery with paclitaxel further potentiates dofetilide-mediated antitumor activity, indicating that nanogel-mediated K^+^ channel blockade can be combined with chemotherapy to strengthen tumor cell killing [31].

Beyond controlling where the blocker is released, some nanomedicines use K^+^ channel inhibition to interrupt defined tumor survival programs. ApoE peptide-modified liposomes encapsulating the NIR hERG/K_V_11.1 probe A8 (ApoE-Lipo@A8) deliver a functional hERG inhibitor to orthotopic glioblastoma. A8 links a hERG-binding ligand to IR780, enabling channel binding, hERG K^+^ current inhibition and channel-associated fluorescence. ApoE liposomes improve brain-tumor delivery, allowing A8 to act within hERG-positive GBM tissues. After reaching GBM cells, A8 reduces membrane-localized hERG and promotes ER retention, triggering ER stress. This suppresses the CDK2-pRB-E2F axis and induces apoptosis and autophagy, thereby inhibiting intracranial tumor growth, reducing Ki67, increasing TUNEL positivity, prolonging survival and enabling hERG-positive tumor imaging [32]. In addition, TRAM@PPF nanoparticles deliver TRAM-34, a selective K_Ca_3.1 inhibitor, through PEG2000-folate-modified PLGA for pancreatic cancer therapy. K_Ca_3.1 is a calcium-activated K^+^ channel that maintains membrane hyperpolarization and provides the electrochemical drive for sustained Ca^2+^ influx, thereby supporting PDAC cell cycle progression, proliferation, migration and invasion. The folate-modified PLGA carrier improves TRAM-34 solubility, stability, cellular uptake and acidic tumor-associated release. Released TRAM-34 suppresses K_Ca_3.1-associated K^+^ channel signaling, weakening the membrane-potential-dependent support for pancreatic cancer growth. TRAM@PPF decreases PANC-1 viability, enhances apoptosis, suppresses pancreatic tumor growth, reduces tumor weight, prolongs survival and shows favorable biosafety [33].

K^+^ channel blockade also intersects with antitumor immunity when extracellular K^+^ helps maintain an immunosuppressive TAM state. Disulfide-rich mesoporous silica modified with 18-crown-6 ether and loaded with ML133, then coated with DiR-embedded macrophage membranes (D-C/M@CM/DiR), combines extracellular K^+^ capture with K_ir_2.1 inhibition. 18-crown-6 complexes K^+^ released by dying tumor cells, weakening the high-K^+^ signal that favors M2-like TAM polarization, while released ML133 inhibits K_ir_2.1 on macrophage membranes. This dual regulation disrupts K^+^-dependent maintenance of the immunosuppressive TAM phenotype, increases CD80, decreases CD206, promotes M1-like TAM repolarization, enhances CD8^+^ T cell infiltration and suppresses 4T1 tumor growth [34].

### RNAi-mediated channel silencing

When tumor-promoting channel activity depends on sustained channel expression, RNAi-loaded nanomaterials can suppress the channel at the transcript level rather than acutely blocking conductance. DMPC single-lipid nanoparticles deliver siRNAs against *SCN5A*-encoded Na_V_1.5 and its cancer-associated neonatal isoform nNa_V_1.5 in triple-negative breast cancer (TNBC). Silencing Na_V_1.5/nNa_V_1.5 reduces channel expression and attenuates sodium channel-associated eukaryotic elongation factor-2 kinase (EF2K) signaling, thereby weakening downstream integrin β1/Src/FAK, PI3K/Akt, ERK, c-myc and cyclin pathways. This expression-level channel regulation decreases proliferation, colony formation, migration and invasion, while inducing G1 arrest and apoptosis. Systemic nanoparticle delivery suppresses orthotopic TNBC growth and reduces lung metastasis **(**Fig. 2C**)** [35].

### Channel-guided drug delivery

Ion channel expression can also be used to guide nanomedicine entry into tumor cells. Anti-K_V_11.1-functionalized gold nanocarriers use aberrant hERG1/K_V_11.1 expression as a tumor-recognition handle for doxorubicin (DOX) delivery in pancreatic cancer cells. Polyethylene glycol (PEG)-gold nanoparticles (AuNPs) and PEGylated Au(III)-DOX nanoparticles both exploit antibody-mediated K_V_11.1 recognition to enhance nanocarrier internalization, lysosomal trafficking and tumor cell drug exposure. In the Au(III)-DOX system, metal-drug complexation further stabilizes the payload, increases drug loading and supports pH-dependent release. These K_V_11.1-guided formulations increase nuclear DOX accumulation, enhance cell cycle blockade and cytotoxicity, and improve chemotherapeutic efficacy without directly inhibiting ion channel conductance [36, 37].

For K^+^/Na^+^ channel nanomedicine, the clearest mechanistic evidence currently comes from localized channel blockade and RNAi-mediated channel silencing, where channel inhibition or knockdown can be directly linked to apoptosis, mitochondrial dysfunction, immune remodeling or reduced metastasis. In contrast, piezoelectric stimulation and K_V_11.1-guided drug delivery are better viewed as channel-associated bioelectric modulation or channel-guided targeting, rather than direct conductance regulation. Future studies should strengthen electrophysiological validation, tumor–normal selectivity and repeated-dose safety assessment before these strategies can be translated further.

## Nano-enabled calcium channel flux programming in cancer

Ca^2+^ flux is both an ionic event and an intracellular signal, allowing calcium channels to translate membrane-level stimulation into organelle stress, mitochondrial dysfunction, apoptosis, altered migration, immune activation and therapeutic resistance [38, 39]. In nanomedicine, this makes Ca^2+^ homeostasis a flexible intervention point: materials can increase or suppress channel activity, alter channel expression or augment local Ca^2+^ availability, while light, ultrasound, acidity, redox reactions and mechanical stress control where and when Ca^2+^ entry or release occurs [15]. Once cellular buffering capacity is exceeded, this adaptive signal is converted into Ca^2+^ overload and drives tumor cell injury [40].

### Increasing calcium channel activity

Because calcium channels sit at the interface between extracellular stimulation and intracellular calcium stress, they provide a direct handle for amplifying tumor-localized inputs. Channel-activating nanomaterials exploit this interface by bringing agonists, responsive photothermal agents, redox-active components or mechanically active structures close to calcium-permeable channels. Once the induced calcium signal exceeds the buffering capacity of tumor cells, it can drive mitochondrial dysfunction, apoptosis, autophagy disruption, immune remodeling or metastatic suppression.

#### Agonist- or stimulus-driven channel activation

The most direct form of channel activation is to control when and where a gating signal reaches the channel. In CAP-loaded photothermal nanoplatforms, NIR irradiation localizes agonist release near tumor cell membranes, allowing repeated TRPV1 opening to accumulate into Ca^2+^ overload. Semiconducting polymer photothermal nanoagonists encapsulating CAP remotely activate TRPV1 in TRPV1-positive cancer cells. NIR irradiation heats the semiconducting polymer nanoparticle (SPN) lipid shell and repeatedly releases CAP near the cell membrane, driving transient TRPV1 opening and cumulative Ca^2+^ overinflux. Sustained Ca^2+^ loading disrupts mitochondrial V_mem_, promotes Cyt-C release and activates caspase-3-dependent apoptosis. This ion-specific, light-gated mechanism suppresses TRPV1-positive tumors while sparing TRPV1-negative cells at low systemic CAP dosage [9]. Similarly, CAP-decorated semiconducting polymer nanoparticles (CSPN) add photodynamic stress to TRPV1-gated Ca^2+^ overload without requiring exogenous calcium delivery. Under NIR irradiation, the semiconducting polymer generates singlet oxygen to trigger CAP release while inducing photodynamic stress. Released CAP activates TRPV1 channels on cancer cells, driving Ca^2+^ influx and intracellular Ca^2+^ overload. This biomaterial-mediated coupling of ion channel activation with photodynamic therapy (PDT) promotes tumor cell death and supports NIR-II fluorescence imaging-guided cancer treatment [14]. Beyond direct tumor cell Ca^2+^ overload, CAP-loaded manganese oxide (MnO_2_)/BSA/polydopamine (PDA) (MBD&C) NPs deliver a TRPV1 agonist to tumor cells and tumor-innervating sensory fibres. CAP activates TRPV1 channels on tumor membranes, driving Ca^2+^ influx and endogenous Ca^2+^ overload for antitumor immune activation. In sensory nerve terminals, TRPV1-mediated ablation reduces calcitonin gene-related peptide (CGRP) and substance P release, relieving tumor-associated pain and weakening neurogenic tumor-promoting signals. MnO_2_ further alleviates hypoxia and mitigates the immunosuppressive tumor microenvironment [41]. Agonist-loaded nanocarriers can also use calcium channel activation to regulate migration and immune remodeling rather than only tumor cell killing. WS12-loaded PEG600 lipid nanocapsules (LNC600-WS12) stimulate TRPM8 activity at the plasma membrane, engaging a channel state associated with reduced prostate cancer cell migration. This biomaterial-mediated TRPM8 activation suppresses migratory behavior in vitro and limits prostate cancer cell dissemination in zebrafish xenografts [42]. Icilin NPs shift TRPM8 agonist-mediated regulation from migration restraint toward Ca^2+^ influx-associated apoptotic stress and immune remodeling. This ion channel activation remodels tumor-derived exosomes, enriching them with immunogenic damage-associated molecular patterns (DAMPs) such as high mobility group box 1 (HMGB1) and HSP70. The resulting exosomes enhance CD4^+^ and CD8^+^ T cell infiltration, shift cytokine signaling toward interleukin-2 (IL-2) and IL-12, suppress IL-10 and PD-L1, and restrain tumor growth and lung metastasis [43].

Channel opening can also be driven by light-responsive physical stimulation rather than agonist release. Biocompatible AQ4N/black phosphorus quantum dot clusters@liposomes (AQ4N/BPCs@Lip) nanocomplexes use membrane-localized photothermal heating to regulate Ca^2+^ influx through thermosensitive TRPV1 channels on tumor cell membranes. Metabolic glycan labelling anchors the liposomal nanocomplexes at the membrane, where 808 nm irradiation enables black phosphorus clusters to generate local heat and open TRPV1 without genetic channel engineering. TRPV1-mediated Ca^2+^ overinflux depolarizes mitochondria and promotes Cyt-C release, while heat-triggered liposome transition releases AQ4N for hypoxia-activated chemotherapy. Coordinated Ca^2+^ overload and AQ4N cytotoxicity intensify apoptosis and suppress MCF-7 tumor growth [44]. CuS-based photothermal NPs further couple heat-triggered channel opening with photoacoustic imaging. Local heating opens TRPV1 channels, driving Ca^2+^ influx and establishing artificial calcium-overload stress in tumor cells independent of hypoxia. The resulting Ca^2+^ imbalance induces mitochondrial dysfunction, marked by caspase-3 and Cyt-C activation, Bcl-2 suppression and ATP depletion. Released CuS nanoparticles further enable photoacoustic imaging, coupling ion channel-regulated tumor killing with diagnostic readout [45]. Membrane anchoring makes this thermal input more spatially confined. TRPV1 antibodies anchor DOX-loaded, PDA-coated hollow Prussian blue nanocages (hPBNCs) on TRPV1-positive cancer cell membranes for membrane-localized TRPV1 activation and chemotherapy. Upon controlled NIR irradiation, local heating opens TRPV1 channels and drives excessive Ca^2+^ influx without bulk hyperthermia. Acidic intracellular exposure of hPBNCs converts endogenous H_2_O_2_ into hydroxyl radicals, while DOX adds chemotherapeutic pressure. TRPV1-mediated calcium overload, chemodynamic ROS and chemotherapy intensify cancer cell apoptosis in vitro and in vivo [46]. Light-triggered calcium channel activation can also be directed toward stem-like tumor cells. Photoactive PEGylated carbon nanohorns (PCNHs), conjugated with TRPV2 antibody through NHS-carbodiimide chemistry and combined with genetic targeting, enable NIR-II light-triggered calcium influx through TRPV2 channels in cancer stem cells. This ion channel-mediated Ca^2+^ entry disrupts Wnt/β-catenin signaling, reducing stemness and selectively eliminating cancer cells. The nanomaterial’s photothermal properties, water dispersibility, and rapid light responsiveness facilitate precise, cell-specific activation, linking biomaterial design to ion channel-regulated tumor eradication [47].

**Fig. 3:**
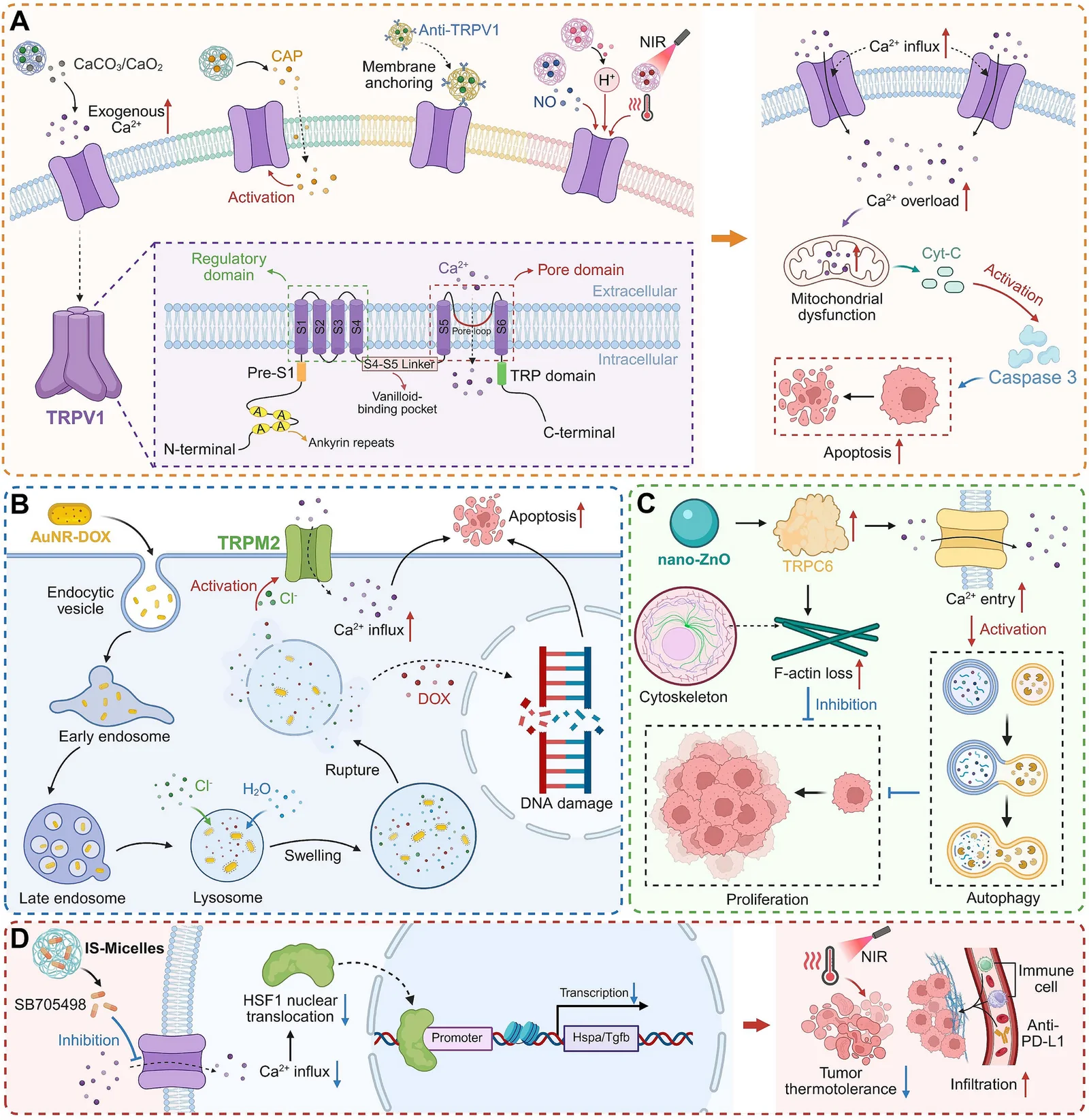
Nanomaterial-mediated regulation of TRP channels in calcium-centered cancer therapy. (**A**) TRPV1-targeted nanoplatforms activate calcium influx through CAP release, exogenous Ca^2+^ supply, anti-TRPV1-mediated membrane anchoring, and NIR-, acid- or NO-responsive channel gating. These strategies amplify Ca^2+^ overload, mitochondrial dysfunction, Cyt-C release, caspase-3 activation and tumor cell apoptosis. (**B**) AuNR-DOX activates TRPM2 through lysosomal proton-sponge rupture and Cl^⁻^ burst-mediated Ca^2+^ influx, while released DOX accumulates in the nucleus and induces DNA damage, jointly promoting apoptosis. (**C**) Nano-ZnO enhances TRPC6-associated Ca^2+^ entry, which is linked to autophagy activation, F-actin loss, cytoskeletal disruption and reduced tumor cell proliferation. (**D**) In IS-Micelles, SB705498 inhibits heat-induced TRPV1-mediated Ca^2+^ influx during mild photothermal therapy, reducing HSF1 nuclear translocation and downstream HSP70 and TGF-β-related transcription. This weakens tumor thermotolerance and improves immune cell and anti-PD-L1 infiltration

Chemical and organelle-localized cues provide another route for stimulus-driven channel activation. At the plasma membrane, TRPV1 antibody-decorated hollow copper sulfide (HCuS)-PDA nanogenerators loaded with BNN6 (HCuS@PDA-TRPV1/BNN6) remotely manipulate TRPV1 signaling through NIR-triggered nitric oxide (NO) release. Upon irradiation, locally generated NO activates TRPV1 channels below the hyperthermic apoptotic threshold, driving excessive Ca^2+^ influx while limiting nonspecific photothermal injury. In parallel, HCuS-mediated chemodynamic reactions amplify oxidative stress. The coupled NO-TRPV1-Ca^2+^ axis and chemodynamic damage intensify cancer cell death with low systemic toxicity [48]. **(**Fig. 3A**)** Confined NO signaling can also be directed to intracellular TRPV1 pools. Tumor membrane-coated, ER-targeted biomimetic upconversion nanoassemblies (M-UCN-T) modulate ER-localized TRPV1 channels in glioblastoma (GBM) through spatially confined NO release. NIR light and glutathione (GSH) trigger in situ NO generation, activating ER-TRPV1 through cysteine nitrosylation. ER-TRPV1 activation initiates Ca^2+^-dependent multi-organelle stress, disrupting ER and downstream organelle homeostasis and driving multimodal GBM cell killing [10]. Nanoparticle-triggered TRP activation can also extend Ca^2+^ entry into broader multi-cation stress. Silver nanoparticles (AgNPs) sensitize HT-29 colorectal cancer cells to 5-fluorouracil (5-FU) through TRPV1-dependent ion channel activation. TRPV1 stimulation enhances cytosolic Ca^2+^ and mitochondrial Ca^2+^/Zn^2+^ accumulation, increasing ROS generation, lipid peroxidation and mitochondrial membrane depolarization. These ion flux-coupled oxidative signals activate caspase-3, -8 and -9, deplete GSH antioxidant defenses, and amplify 5-FU-induced apoptosis and cell death; TRPV1 antagonism attenuates these effects [49]. AgNPs can also potentiate CDDP cytotoxicity through TRPM2 activation. AgNP-induced oxidative stress stimulates TRPM2 activity, promoting Ca^2+^ and Zn^2+^ influx into mitochondria and amplifying mitochondrial depolarization, ROS generation and lipid peroxidation. TRPM2 antagonists attenuate these effects, linking nanoparticle-triggered ion channel activation to caspase-dependent apoptosis. This biomaterial-chemotherapy combination exploits TRPM2-mediated ionic stress to enhance GBM cell killing [50]. TRPM2 can also be activated through lysosomal chloride release rather than AgNP-induced oxidative stress. Lysosomal stress can trigger a different chemical route to channel-mediated Ca^2+^ entry. Cationic cetyltrimethylammonium bromide (CTAB)-coated gold nanorods covalently conjugated with DOX (AuNR-DOX) activate TRPM2 channels through a lysosome-dependent chloride-calcium axis. After endocytosis and endosome-lysosome trafficking, acidic hydrolases release DOX and re-expose the cationic AuNR surface, inducing lysosomal proton-sponge stress, swelling and membrane rupture. The resulting burst release of lysosomal Cl^⁻^ into the cytoplasm stimulates TRPM2-mediated extracellular Ca^2+^ influx, while released DOX accumulates in the nucleus to provide a parallel chemotherapeutic apoptotic signal. This dual mechanism couples DOX-mediated nuclear cytotoxicity with TRPM2-dependent Ca^2+^ influx, increasing apoptotic cancer cell death **(**Fig. 3B**)** [51]. Calcium entry can also be amplified by increasing channel abundance rather than by acutely opening an existing channel. Zinc oxide nanoparticles (nano-ZnO) reshape neuroblastoma Ca^2+^ homeostasis by enhancing TRPC6 expression and TRPC-dependent Ca^2+^ entry. Elevated Ca^2+^ influx increases LC3 puncta formation and autophagic responses without engaging canonical apoptotic markers such as Cyt-C, caspase-3, Bcl-xL or BAX. TRPC inhibition blocks both Ca^2+^ influx and F-actin loss, indicating that nano-ZnO suppresses tumor cell proliferation through TRPC6-mediated calcium dysregulation and cytoskeletal disruption **(**Fig. 3C**)** [52]. Beyond plasma-membrane calcium entry, oxidative stress can also open intracellular calcium-release channels. Snakeberry-like porous *m*-aminophenol-formaldehyde (APF)@ZIF-67 nanoreactors photoactivate endogenous inositol 1,4,5-trisphosphate receptor (IP_3_R) channels by generating H_2_O_2_ under visible light. H_2_O_2_ oxidizes IP_3_R cysteine residues, releasing ER Ca^2+^ that is rapidly transported into mitochondria through mitochondrial Ca^2+^ uniporter (MCU), producing mitochondrial Ca^2+^ overload. Concurrent hydroxyl radical (•OH) generation and mild photothermal heating intensify oxidative stress, ER stress and mitochondrial dysfunction. Persistent Ca^2+^ dyshomeostasis activates pro-apoptotic unfolded protein response (UPR), inflammatory senescence and apoptosis, suppressing tumor growth through endogenous calcium-store mobilization [53].

Mechanical inputs further broaden channel activation by converting membrane tension or nanoparticle stiffness into Ca^2+^ influx. Polymeric nanothreads P-BS-CM1 and P-CM2 self-assemble into cell surface patches that cluster CXC chemokine receptor 4 (CXCR4) and generate mechanical stress on cancer cell membranes. P-BS-CM1 contains a CXCR4-binding sequence and coil motif 1, whereas P-CM2 provides the complementary coil motif 2 for patch formation. This stress activates PIEZO1, linking CXCR4 clustering to mechanically gated calcium signaling. PIEZO1 activation amplifies CXCR4 antagonism, suppresses epithelial-mesenchymal transition programs and reduces metastatic activity, whereas PIEZO1 inhibition weakens the nanothread-induced effect [54]. In macrophages, soft silica NPs use elasticity-dependent membrane deformation to engage PIEZO1 signaling. PIEZO1 activation drives Ca^2+^ influx, NF-κB signaling and inflammatory polarization, converting M2-like tumor-associated macrophages (TAMs) toward an M1 phenotype. The softest nanoparticles enhance tumor penetration and accumulation, repolarize macrophages within hypoxic tumor regions, and suppress tumor growth through mechanically coupled ion channel activation and immune remodeling [55]. Yoda1-loaded, CRV-targeted stiffness-tuned mesoporous silica nanoparticles (YmSNs@CRV), in which Yoda1 acts as a PIEZO1 agonist and CRV serves as a TAM-targeting peptide, further extend macrophage PIEZO1 activation to exosome-mediated T cell stimulation. Softer MSNs more effectively engage PIEZO1, increasing CD80 expression, pro-inflammatory cytokine secretion and exosome production. These macrophage-derived exosomes activate T cell receptor (TCR) signaling, amplify cytotoxic T cell infiltration and reinforce M1-like antitumor polarization. By converting nanoparticle mechanics into PIEZO1-dependent immune reprogramming, YmSNs@CRV suppress tumor growth through coupled macrophage repolarization and exosome-driven immune activation [56].

#### Gene-enabled channel amplification or installation

In addition to gating calcium channels that are already present in tumor cells, nanomaterials can shift calcium channel activity upstream by changing the channel repertoire itself. Through plasmid delivery, these systems either amplify an endogenous calcium-permeable channel or install an engineered mechanosensitive gate, so that subsequent optical or acoustic stimulation produces a more programmable Ca^2+^ influx. For example, NIR-responsive pTRPM2-loaded conjugated oligomer nanoparticles (CONs/pTRPM2) convert 808 nm light into a low-lethality ROS pulse that primes TRPM2-dependent calcium entry in tumor cells. Under 808 nm irradiation, ROS cleaves Se-Se bonds to release a plasmid encoding transient receptor potential melastatin-2 (pTRPM2), increasing TRPM2 expression in tumor cells. Concurrent ROS activation of TRPM2 promotes Ca^2+^ influx, suppresses early autophagy and amplifies intracellular ROS accumulation. The coupled ROS-TRPM2-Ca^2+^ cascade disrupts tumor cell homeostasis and induces cancer cell death under remote optical control [57].

### Suppressing calcium channel activity or expression

Although calcium influx is often exploited as a cytotoxic stress, calcium channel signaling can also help tumors adapt to therapy by sustaining heat-shock responses, anti-apoptotic signaling, chemoresistance and immune escape. In this context, nanomaterials provide a complementary route for calcium regulation: instead of forcing channels open, they deliver blockers or gene-silencing cargos to quiet pro-survival calcium signals.

#### Pharmacological channel blockade

Pharmacological blockade is particularly useful when treatment itself induces protective calcium signaling. During mild photothermal therapy, amphiphilic polypeptide-cyanine fluorescent nanoprobes physically encapsulating the TRPV1 blocker SB705498 (PLFA-SB) inhibit heat-induced TRPV1-mediated Ca^2+^ influx. This blockade suppresses heat shock transcription factor 1 (HSF1) translocation and reduces HSP expression. This weakened heat shock response lowers tumor thermotolerance, increasing photothermal tumor killing under sub-45 °C heating while retaining NIR-II fluorescence imaging capability [58]. In ICG/SB705498-loaded PEG-poly(caprolactone) (PCL) polymeric micelles (IS-Micelles), TRPV1 blockade extends this thermotolerance-control strategy into thermo-immunotherapy. By restricting hyperthermia-induced Ca^2+^ influx, SB705498-mediated TRPV1 inhibition prevents HSF1 nuclear translocation and reduces stress-induced HSP70 expression, weakening thermal resistance. HSF1 suppression also restrains transforming growth factor β (TGF-β) signaling, remodels fibrotic tumor stroma and improves immune cell and anti-PD-L1 infiltration **(**Fig. 3D**)** [59].

Blocker-loaded nanomaterials can also sensitize tumors to cytotoxic therapy by weakening calcium-dependent survival and resistance pathways. Paclitaxel-loaded Pluronic-polyethylenimine gold-caged nanoparticles (PTX-PP@Au NPs) regulate androgen-resistant prostate cancer by combining TRPV6 channel blockade with paclitaxel delivery and NIR-triggered photothermal-photodynamic effects. Channel inhibition perturbs TRPV6-dependent calcium signaling, while paclitaxel reinforces cell cycle arrest. Under single NIR irradiation, the gold cage elevates local temperature and generates ROS, amplifying cytotoxicity and apoptosis. The combined disruption of calcium influx, mitotic progression and redox balance suppresses androgen-resistant prostate tumor growth [60]. In breast cancer, NIR-responsive HC-030031-loaded photothermal conjugated polymer nanoparticles (NPs-H) use heat-triggered blocker release to inhibit ROS-sensitive TRPA1 signaling. Released HC-030031 inhibits TRPA1-associated Ca^2+^ influx, reduces Ca^2+^/calmodulin complex formation and downregulates the anti-apoptotic protein MCL-1. Disruption of TRPA1-mediated calcium signaling weakens oxidative-stress defence, restores apoptotic sensitivity, reduces chemoresistance and suppresses tumor growth in vivo [61].

Channel blockade can also be sustained in hematologic malignancy through biomimetic membrane targeting. In acute myeloid leukaemia (AML), leukemia cell membrane-coated poly(lactic-co-glycolic acid) (PLGA) nanoparticles encapsulating curcumin (CUR) (PLGA-C-M) use homologous membrane mimicry and sustained drug release to inhibit TRPM2 channels in U937 leukemia cells. CUR-loaded PLGA-C-M reduces intracellular Ca^2+^ signaling, lowers ATP production, increases intracellular and mitochondrial ROS, and disrupts mitochondrial membrane potential. This TRPM2-directed blockade also suppresses autophagy and weakens Dox resistance, thereby reducing leukemia cell survival in vitro and inhibiting AML progression in vivo [62]. In some blocker-based designs, suppressing calcium uptake does more than improve cytotoxic sensitivity; it also creates an immunogenic stress signal. pH-responsive Zn-Al layered double hydroxide nanosheets co-loaded with the T-type Ca^2+^ channel inhibitor TTA-Q6 and the CD47 inhibitor RRX-001, termed LRT nanomedicines, release TTA-Q6 in acidic tumor-stromal regions. TTA-Q6 disrupts tumor Ca^2+^ uptake, inducing ER stress and calreticulin exposure on cancer cells. This ion channel blockade activates macrophage phagocytosis and dendritic cell (DC) maturation, while RRX-001 reduces CD47-mediated immune evasion. Coordinated calcium channel inhibition and checkpoint disruption promote antigen presentation, antitumor T cell activation and resistance to tumor re-challenge [63].

#### RNAi-based channel silencing

When sustained suppression of calcium channel signaling is needed, RNAi delivery provides an expression-level alternative to pharmacological blockade. In drug-resistant breast cancer, mesoporous silica nanocapsules (MSNCs) co-delivering DOX and T-type Ca^2+^ channel-specific siRNA (pMSNC/DOX/siRNA) reduce T-type Ca^2+^ channel expression in MCF-7/adriamycin (ADR) cells. siRNA-mediated channel knockdown lowers cytosolic Ca^2+^ levels, inducing G0/G1 arrest and enhancing intracellular drug accumulation in ADR cells. Coupled calcium channel silencing and chemotherapy produce synergistic tumor cell killing in vitro and improved suppression of drug-resistant breast tumors in vivo [64]. Similarly, internalizing RGD (iRGD)-modified lipid-coated silica NPs co-delivering Ca^2+^ channel siRNA with ADR (LSC/R-A) downregulate T-type Ca^2+^ channels in multidrug-resistant liver cancer. Channel silencing lowers cytosolic Ca^2+^, arrests HepG2/ADR cells at G0/G1 phase and increases intracellular drug retention. This LSC/R-A-mediated regulation of calcium channel expression reverses multidrug resistance (MDR) and enhances ADR-mediated tumor inhibition compared with single-agent siRNA or chemotherapy [65].

Beyond T-type channel knockdown, siRNA delivery can also target broader calcium-associated survival pathways. HA-hybridized carbonate apatite nanoparticles provide a biomaterial platform for siRNA delivery against ion channel-associated cancer signaling. By strengthening siRNA binding, enhancing cellular uptake and limiting off-target biodistribution, HA-carbonate apatite (CA) NPs efficiently silence *TRPC6*, *TRPM8* and *SLC41A1* in breast cancer cells. Suppression of these calcium channel and transporter genes disrupts Ca^2+^-dependent tumor survival pathways, producing enhanced cytotoxicity in vitro and improved breast tumor targeting in vivo [66]. A related carbonate apatite platform broadens this RNAi-based approach by targeting multiple Ca^2^⁺ channels and Mg^2^⁺ transporters simultaneously. Its Ca^2+^-rich domains bind anionic siRNAs, promote endocytic uptake and support cytosolic release after pH-responsive dissolution. Knockdown of *TRPC6*, *TRPM7*, *TRPM8*, *ORAI1/3* and *ATP2C1* perturbs Ca^2+^ signaling, while *SLC41A1/2* and *MAGT1* silencing disrupts Mg^2+^ transport. Combined ion-transport gene suppression reduces Akt signaling and compromises breast cancer cell viability [67].

### Augmenting calcium availability

Calcium overload can also be initiated from the calcium pool rather than from a defined channel target. In calcium-supplementing systems, nanomaterials increase local Ca^2+^ availability, which can then engage calcium-responsive channels and receptors more broadly. Dendritic mesoporous bioactive glass (MBG) nanospheres release extracellular Ca^2+^ to activate TRP channels together with calcium-sensing receptors on tumor cells. The resulting calcium influx engages the calpain-1-Bcl-2-caspase-3 pathway, selectively suppressing tumor growth while sparing normal cells. Their dendritic mesoporous architecture further enables high drug loading and controlled release, prolonging drug action and improving antitumor efficacy with reduced systemic toxicity [68].

### Mixed calcium channel and calcium-availability mechanisms

In many calcium-overload platforms, channel activation and calcium supplementation are designed to reinforce one another. Rather than relying only on channel gating or calcium supplementation, the same nanoplatform may open calcium-permeable channels while also providing an additional calcium source, mobilizing intracellular stores or limiting calcium clearance. These coupled inputs make calcium stress more sustained than either channel gating or calcium supplementation alone.

#### Channel activation plus exogenous calcium supply

The most direct coupling pairs channel gating with a local calcium source, allowing exogenous Ca^2+^ release to reinforce channel-mediated Ca^2+^ entry. In acid-responsive calcium carbonate (CaCO_3_) nanoparticles, released CAP activates TRPV1 channels and drives Ca^2+^ influx across the tumor cell membrane, while CaCO_3_ dissolution increases local calcium availability. The combined contribution of exogenous Ca^2+^ release and channel-mediated Ca^2+^ entry disrupts intracellular calcium homeostasis, producing sustained Ca^2+^ overload. This calcium dyshomeostasis activates apoptotic signaling and induces tumor cell death [13]. Similarly, pH-responsive CAP/bovine serum albumin (BSA)@ tricalcium phosphate (TCP)-zeolitic imidazole framework-8 (ZIF-8) NPs use acidic tumor degradation to release CAP and Ca^2+^ for TRPV1-gated Ca^2+^ overload. In acidic tumor environments, nanoparticle degradation supplies exogenous Ca^2+^ and releases CAP, which activates TRPV1 channels to promote further Ca^2+^ influx. The combined channel-mediated entry and exogenous Ca^2+^ delivery disrupt calcium homeostasis, damage mitochondria and trigger apoptosis in tumor cells [69]. This CAP-gated design can be further coupled with catalytic oxidative stress. Thermo/acid-responsive CaAuMn-CAP@PEG nanoreactors (CAMcaP) use laser-triggered shell disassembly and acidic CaAuMn degradation to drive TRPV1-gated Ca^2+^ overload in lung adenocarcinoma (LUAD). Released CAP opens membrane TRPV1 channels, while CaAuMn supplies Ca^2+^; Mn^2+^-mediated Fenton-like catalysis, accelerated by photothermal heating, generates a ROS storm. The convergent Ca^2+^ influx and oxidative stress disrupt mitochondrial homeostasis and ablate tumors, with CaAuMn supporting fluorescence/computed tomography (FL/CT) imaging [70].

Channel activation can also be coupled to calcium release through material-generated heat or acidity rather than CAP-mediated agonist gating. NIR-responsive NPs-PCa integrate photothermal TRPV1 activation, CaO_2_-derived calcium release and DOX delivery to amplify intracellular Ca^2+^ stress in breast cancer cells. Upon irradiation, local heating triggers Ca^2+^ release from the nanoparticle core and activates the TRPV1 channels, enabling extracellular Ca^2+^ influx. The combined nanoparticle-derived Ca^2+^ release and channel-mediated entry elevate cytosolic Ca^2+^, induce mitochondrial dysfunction and promote cancer cell death through photothermally controlled Ca^2+^ overload [71]. Similarly, a multifunctional nanoparticle loading CaCO_3_, 2Br-NDIA and quercetin (CNQ) uses mild photothermal heating to activate TRPV1 channels while supplying additional Ca^2+^ in hepatocellular carcinoma (HCC). Upon irradiation, CNQ activates TRPV1 channels to promote Ca^2+^ influx, while CaCO_3_ supplies additional Ca^2+^. The coupled channel activation and calcium release disrupt intracellular Ca^2+^ homeostasis, drive mitochondrial Ca^2+^ overload, weaken tumor self-defence and induce cancer cell death under mild photothermal conditions [72]. Ferric PDA NPs mineralized with a CaCO_3_ nanoshell (Fe-PDA@CaCO_3_ NPs) combine calcium storage, photothermal activation and chemodynamic stress. In acidic tumor microenvironments, the CaCO_3_ shell releases Ca^2+^, while NIR-heated Fe-PDA activates TRPV1 channels to enhance cytoplasmic Ca^2+^ influx. Concurrent Fe-mediated hydroxyl radical generation suppresses Ca^2+^ efflux, sustaining intracellular calcium accumulation. The combined Ca^2+^ overload and ROS elevation disrupt mitochondrial homeostasis and activate apoptosis in tumor cells [73]. Photoacidification provides another route to the same coupled input. NIR-activated upconversion nanoparticle (UCNP)-engineered covalent organic framework (COF) nanocomposites@CaO_2_-hyaluronic acid (HA)/photoacid generator (PAG)/indocyanine green (ICG) (UCCPI) photoacidify cancer cells to activate acid-sensitive TRPV1 channels. Under 980 nm irradiation, photoacid generators release H^+^, promoting TRPV1-dependent Ca^2+^ influx and accelerating HA-functionalized CaO_2_ decomposition for additional Ca^2+^ supply. CaO_2_-derived O_2_ supports indocyanine-green-mediated ROS generation, weakening mitochondrial calcium buffering. TRPV1-driven Ca^2+^ entry, CaO_2_-derived calcium release and PDT-induced oxidative stress induce mitochondrial dysfunction and apoptotic tumor cell death [74].

#### Channel activation with intracellular calcium release

Channel-driven calcium stress can also be intensified by coupling membrane Ca^2+^ entry with calcium release from intracellular stores. HA-modified, CUR-loaded hollow mesoporous Prussian blue nanoparticles (HCPH NPs) use acid-triggered CUR release to mobilize endoplasmic reticulum (ER) Ca^2+^ and suppress heat shock protein 90 (HSP90), while NIR irradiation opens membrane TRPV1 channels. The resulting dual calcium input drives cytoplasmic and mitochondrial Ca^2+^ overload, causing mitochondrial dysfunction, ATP depletion and weakened thermotolerance during photothermal tumor ablation [75]. In I/B@MSN-T@HA, this membrane–ER coupling is driven by paired ROS and NO signals. The HA-gated mesoporous silica nanomodulator, based on TPP-functionalized mesoporous silica nanoparticles co-loaded with ICG and the heat-sensitive NO donor BNN-6, uses light-triggered ROS to activate TRPA1 channels and promote extracellular Ca^2+^ influx. Meanwhile, photothermal decomposition of BNN-6 releases NO that opens ER ryanodine receptors. The synchronized Ca^2+^ entry and ER release drive cytoplasmic calcium overload, disrupt calcium homeostasis and inhibit tumor growth [76].

#### Calcium supply with efflux blockade or nonspecific channel dysregulation

**Fig. 4:**
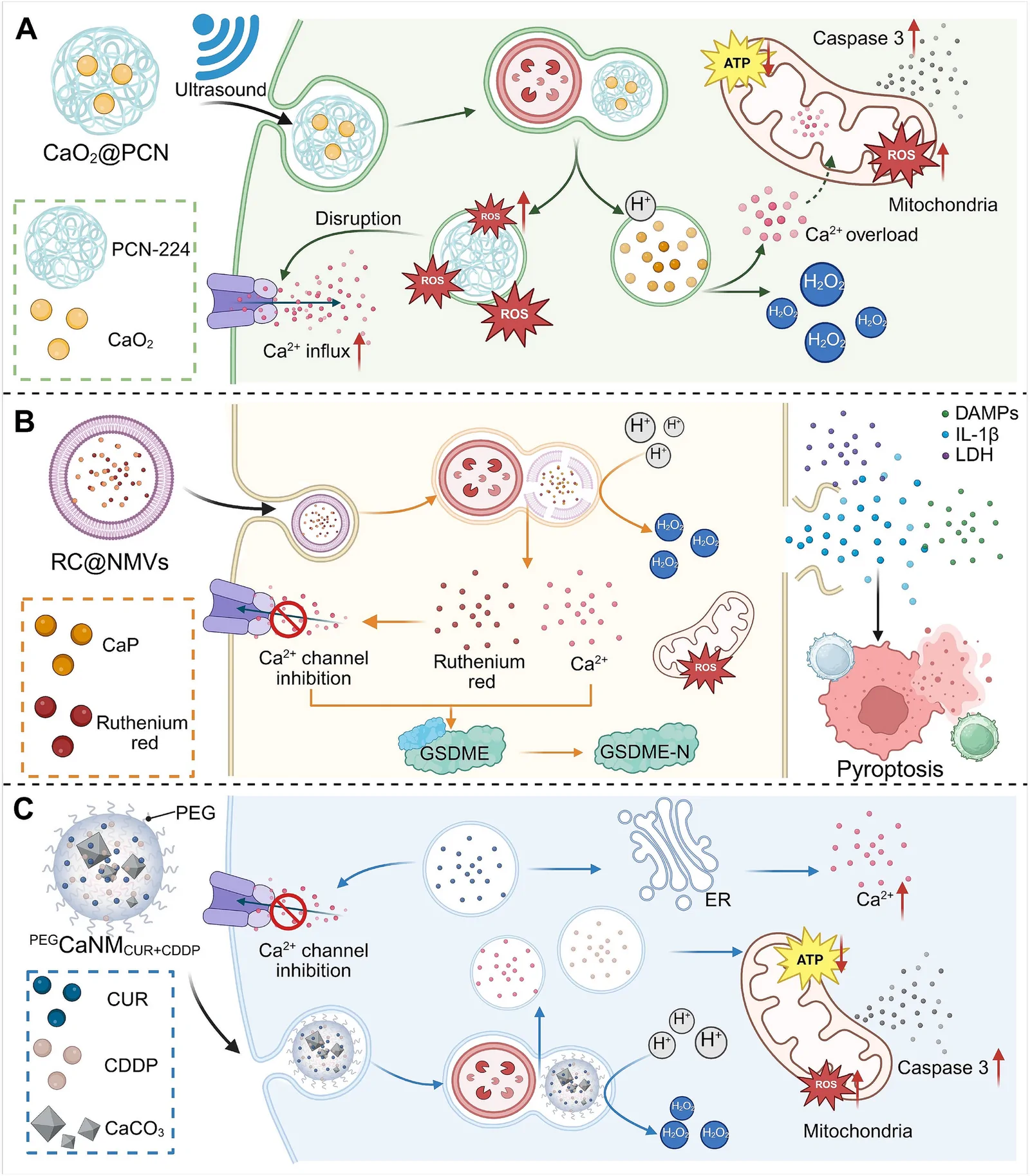
Nanomaterial-mediated amplification of calcium overload in calcium-centered cancer therapy. (**A**) Ultrasound-activated CaO_2_@PCN generates ROS and releases Ca^2+^/H_2_O_2_ after lysosomal degradation, amplifying calcium influx, calcium overload, mitochondrial dysfunction and tumor cell death. (**B**) RC@NMVs disrupt tumor calcium homeostasis through CaP-derived Ca^2+^ release and ruthenium red-mediated calcium channel blockade, triggering gasdermin-dependent pyroptosis and inflammation-amplified antitumor immunity. (**C**) ^PEG^CaNM_CUR+CDDP_ nanoparticles promote Ca^2+^ release, inhibit calcium efflux and deliver cisplatin, inducing progressive calcium dysregulation and mitochondrial damage in cancer cells

When calcium release is combined with impaired calcium clearance or nonspecific channel dysregulation, exogenous Ca^2+^ supply can evolve from simple supplementation into a self-amplifying disturbance of calcium homeostasis. Calcium peroxide coated with porphyrinic metal–organic framework (MOF) porous coordination network-224 (PCN-224) (CaO_2_@PCN) uses ultrasound-responsive ROS generation and CaO_2_ degradation for sonodynamic ion interference. Under ultrasound, PCN-224 generates ROS and promotes abnormal calcium channel activity, while acoustic stimulation enhances extracellular Ca^2+^ influx. Lysosomal degradation of CaO_2_@PCN releases Ca^2+^ and H_2_O_2_, further amplifying calcium channel dysregulation, intracellular Ca^2+^ overload and persistent ROS production. The coupled oxidative stress and ion imbalance induce mitochondrial dysfunction and tumor cell death **(**Fig. 4A**)** [77].

This calcium disturbance becomes self-amplifying in PEG-modified calcium phosphate (CaP)-coated, manganese-doped hollow mesoporous Prussian blue nanoparticles encapsulating glucose oxidase (GMCP), which supply exogenous Ca^2+^ while restricting Ca^2+^ efflux. CaP degradation releases exogenous Ca^2+^, while Fenton/Fenton-like reactions, GSH depletion and glucose oxidase-mediated starvation elevate intracellular ROS. The resulting oxidative stress damages mitochondria and suppresses Ca^2+^ efflux channels, sustaining intracellular calcium accumulation. This reciprocal ROS-Ca^2+^ amplification drives mitochondrial dysfunction and apoptotic death in tumor cells [78]. In addition, neutrophil membrane-camouflaged CaP NPs co-loaded with ruthenium red (RC@NMVs) exploit tumor inflammation to disrupt calcium homeostasis. Lysosomal dissolution of the CaP core releases Ca^2+^, while ruthenium red blocks Ca^2+^ transporting channels, inducing intracellular Ca^2+^ overload that triggers gasdermin-mediated pyroptosis. The resulting release of DAMPs and cytokines amplifies the inflammatory microenvironment, enhancing nanoparticle tumor accumulation and activating durable antitumor immunity **(**Fig. 4B**)** [79]. CaCO_3_-based carriers can also turn calcium release into a combined drug-delivery and imaging platform. PEGylated CaCO_3_ nanoparticles templated by polydopamine and co-loaded with CUR and CDDP (^PEG^CaNM_CUR+CDDP_) selectively accumulate in tumors and amplify calcium-overload-mediated mitochondrial dysfunction. After tumor accumulation and cellular uptake, burst Ca^2+^ release from CaCO_3_ is combined with CUR-mediated inhibition of Ca^2+^ efflux and CDDP cytotoxicity, producing multichannel calcium dysregulation and progressive mitochondrial damage. CUR fluorescence and PDA-based photoacoustic contrast further enable imaging-guided tumor targeting **(**Fig. 4C**)** [80]. In these systems, calcium overload is better understood as a loss of buffering capacity produced by converging stresses, rather than as the output of one channel alone. **(**Table 1**).**

**Table 1:** Nanoplatforms targeting Ca^2+^ channels and calcium homeostasis for cancer therapy

Nanoplatform	Ca^2+^ channel	Channel regulation	Ca^2+^ flux alteration	Tumor model	Therapeutic outcome	Ref
CAP-encapsulated SPN	TRPV1	NIR-triggered CAP release; repeated channel activation	Ca^2+^ overinflux ↑; mitochondrial ion overload ↑	TRPV1^+^ U373 and TRPV1^—^ HeLa tumors	Mitochondrial apoptosis ↑; TRPV1^+^ tumor growth ↓ (not TRPV1^−^)	[9]
CSPN		NIR-triggered CAP release; PDT coupling	Ca^2+^ influx ↑; Ca^2+^ overload ↑	U373 GBM	PDT efficacy ↑; tumor imaging enabled; tumor growth ↓	[14]
CAP-loaded CaCO_3_ NPs		Acid-triggered CAP/Ca^2+^ release; channel activation	Ca^2+^ influx ↑; Ca^2+^ overload ↑	HepG2 HCC	Apoptosis ↑; tumor growth ↓	[13]
CAP/BSA@TCP-ZIF-8 NPs		Acid-triggered CAP/Ca^2+^ release; CAP activation	Ca^2+^ influx ↑; Ca^2+^ overload ↑	HepG2 HCC	Mitochondrial damage and apoptosis ↑; tumor growth ↓	[69]
CAMcaP		Thermo/acid-triggered CAP release; ROS storm	Ca^2+^ influx ↑; ROS-Ca^2+^ stress ↑	LUAD	Mitochondrial dysfunction ↑; tumor ablation ↑; enabling FL/CT imaging	[70]
MBD&C NPs		CAP activation; TRPV1^+^ terminal ablation	Ca^2+^ influx ↑; endogenous Ca^2+^ overload ↑	4T1 breast cancer	Tumor growth ↓; cancer pain ↓; antitumor immunity ↑	[41]
AQ4N/BPCs@Lip nanocomplexes		Membrane-localized photothermal gating	Ca^2+^ overinflux ↑	MCF-7 breast cancer	Mitochondrial apoptosis ↑; hypoxia-activated AQ4N chemotherapy ↑	[44]
NPs-PCa		NIR photothermal gating; CaO_2_ release	Ca^2+^ release ↑; Ca^2+^ influx ↑	4T1 xenograft breast cancer	Mitochondrial dysfunction ↑; tumor growth ↓	[71]
CNQ		Mild photothermal activation; CaCO_3_ supply	Ca^2+^ influx ↑; mitochondrial overload ↑	HepG2 HCC	Mitochondrial Ca^2+^ overload ↑; mild PTT efficacy ↑	[72]
Fe-PDA@CaCO_3_ NPs		NIR photothermal activation; ROS generation	Ca^2+^ influx ↑; Ca^2+^ efflux ↓	HepG2 HCC	Mitochondrial apoptosis ↑; PTT/CDT efficacy ↑	[73]
UCCPI nanomodulators		NIR photoacidification; CaO_2_ decomposition	Ca^2+^ influx ↑; mitochondrial overload ↑	4T1 breast cancer	Oxidative damage ↑; mitochondrial apoptosis ↑	[74]
HCPH NPs		NIR photothermal activation; ER Ca^2+^ mobilization	Ca^2+^ influx ↑; ER Ca^2+^ release ↑	B16-F10 tumor	Thermotolerance ↓; PTT efficacy ↑	[75]
CuS-based photothermal NPs		Mild-acidity/NIR photothermal gating	Ca^2+^ influx ↑; overload ↑	HepG2 HCC	Mitochondrial apoptosis ↑; enabling PA imaging	[45]
M-UCN-T		NIR/GSH-triggered NO release; channel nitrosylation	ER Ca^2+^ stress ↑; multi-organelle Ca^2+^ dysregulation ↑	GBM	Multi-organelle stress ↑; tumor cell killing ↑	[10]
HCuS@PDA-TRPV1/BNN6		NIR-triggered NO release; channe activation	Ca^2+^ influx ↑; overload ↑	U373 GBM	Cancer cell death ↑; CDT efficacy ↑	[48]
DOX-loaded hPBNCs		TRPV1-antibody anchoring; NIR photothermal activation	Ca^2+^ influx ↑; overload ↑	U373 GBM	Apoptosis ↑; chemotherapy/CDT efficacy ↑	[46]
PLFA-SB		SB705498 blockade; NIR-II photothermal heating	Ca^2+^ influx ↓; HSF1/HSP signaling↓	4T1 breast cancer	Thermotolerance ↓; mPTT efficacy ↑; NIR-II imaging enabled	[58]
IS-Micelles		SB705498 blockade; NIR photothermal activation	Heat-induced Ca^2+^ influx ↓	Breast, liver, colorectal and pancreatic tumors	Thermoresistance ↓; tumor growth ↓; anti-PD-L1 and immune cell infiltration ↑; immune memory ↑	[59]
PCNHs	TRPV2	NIR-II photothermal activation; genetic targeting	Ca^2+^ influx ↑	A549 lung cancer	Cancer stemness ↓; Wnt/β-catenin signaling ↓	[47]
PTX-PP@Au NPs	TRPV6	Channel blockade; PTX delivery; NIR PTT/PDT	Ca^2+^ signaling ↓	Androgen-resistant prostate cancer	Apoptosis ↑; tumor growth ↓; multimodal therapy efficacy ↑	[60]
AuNR-DOX	TRPM2	Lysosomal rupture; Cl⁻ burst-driven activation	Ca^2+^ influx ↑	H1975 LUAD	Cancer cell apoptosis ↑	[51]
CONs/pTRPM2		ROS generation; pTRPM2 release; channel activation	Ca^2+^ influx ↑; ROS-Ca^2+^ amplification ↑	PC-3 prostate cancer	Early autophagy ↓; tumor therapy efficacy ↑	[57]
PLGA-C-M		Leukemia membrane targeting; sustained stepwise regulation	ROS-Ca^2+^ disturbance ↑	AML	Mitochondrial dysfunction ↑; autophagy ↓; drug resistance ↓	[62]
LNC600-WS12	TRPM8	WS12 delivery; channel activation	Intracellular Ca^2+^ ↑	PC-3 prostate cancer	Tumor cell migration ↓; zebrafish xenograft dissemination ↓	[42]
Icilin NPs		Icilin activation; cold channel stimulation	Ca^2+^ influx ↑	4T1 breast cancer	Immunogenic exosomes ↑; tumor growth/metastasis ↓	[43]
I/B@MSN-T@HA	TRPA1 / RyR axis	ROS-activated TRPA1; NO-triggered RyR opening	Ca^2+^ influx ↑; ER Ca^2+^ release ↑; Ca^2+^ overload ↑	4T1 breast cancer	Tumor growth ↓	[76]
NPs-H	TRPA1	NIR-mediated TRPA1 inhibition	Ca^2+^ influx ↓; Ca^2+^/calmodulin signaling ↓	HCC1569 breast cancer	Chemoresistance ↓; tumor growth ↓	[61]
Nano-ZnO	TRPC6	TRPC6 expression ↑; TRPC activation	Ca^2+^ entry ↑	Neuroblastoma	Autophagy ↑; proliferation ↓	[52]
HA-CA NPs	TRPC6 / TRPM8 / SLC41A1	siRNA-mediated gene silencing	Ca^2+^-dependent survival signaling ↓	MCF-7 breast cancer	Cytotoxicity ↑; breast tumor targeting ↑	[66]
Dendritic MBG nanospheres	TRP channels / CaSR	Ca^2+^ release; TRP/CaSR activation	Ca^2+^ influx ↑	S180 sarcoma	Calpain-1–Bcl-2–caspase-3 signaling ↑; tumor growth ↓	[68]
LRT nanomedicines	T-type Ca^2+^ channel	TTA-Q6 blockade; CD47 inhibition	Ca^2+^ uptake ↓; ER stress ↑	Lung cancer	Antigen presentation ↑; antitumor immunity ↑	[63]
pMSNC/DOX/ siRNA		siRNA knockdown; DOX co-delivery	Cytosolic Ca^2+^ ↓	MCF-7/ADR breast cancer	MDR ↓; drug accumulation ↑; tumor inhibition ↑	[64]
iRGD-LSC/R-A		siRNA knockdown; ADR co-delivery	Cytosolic Ca^2+^ ↓	Multidrug-resistant liver cancer	MDR ↓; ADR efficacy ↑	[65]
P-BS-CM1/P-CM2 nanothread patches	PIEZO1	CXCR4 clustering; mechanotransduction	Mechanically gated Ca^2+^ signaling ↑	4T1 breast cancer	EMT/metastasis ↓; CXCR4 antagonism ↑	[54]
Soft silica NPs		Elasticity-dependent mechanical activation	Ca^2+^ influx ↑; NF-κB signaling ↑	4T1 breast cancer	TAM M1 polarization ↑; tumor growth ↓	[55]
YmSNs@CRV		Yoda1 delivery; stiffness-tuned activation	Ca^2+^ influx ↑; exosome signaling ↑	4T1 and CT26 tumors	TAM reprogramming ↑; T cell infiltration ↑	[56]
MscL plasmid-loaded cationic nanoliposomes	MscL I92L	Ultrasound-gated channel opening	Ca^2+^ influx ↑; Ca^2+^ apoptosis pathway ↑	B16 tumor	Tumor cell apoptosis ↑	[11]
FeAlg/PEI/MSCL	MscL	Ultrasound-triggered channel opening	Sustained Ca^2+^ influx ↑	Melanoma	Tumor cell apoptosis ↑; antitumor immunity ↑	[89]
APF@ZIF-67 nanoreactors	IP_3_R	H_2_O_2_-mediated IP_3_R oxidation	ER Ca^2+^ release ↑; mitochondrial overload ↑	A549 lung cancer	Mitochondrial dysfunction ↑; apoptosis ↑	[53]
CaO_2_@PCN	Ca^2+^ channels	SDT-induced abnormal channel activity	Ca^2+^ influx ↑; Ca^2+^ overload ↑	4T1 breast cancer	ROS generation ↑; mitochondrial dysfunction ↑	[77]
GMCP	Ca^2+^ efflux channels	CaP degradation; ROS-amplified efflux suppression	Ca^2+^ overload ↑; Ca^2+^ efflux ↓	CT26 tumor	ROS outbreak ↑; oncotherapy efficacy ↑	[78]
RC@NMVs	Ca^2+^ transporting channels	CaP dissolution; ruthenium-red blockade	Ca^2+^ accumulation ↑; Ca^2+^ overload ↑	CT26 tumor	Pyroptosis ↑; antitumor immunity ↑	[79]
^PEG^CaNM_CUR+CDDP_	Ca^2+^ channels	CaCO_3_ degradation; CUR-mediated efflux inhibition	Ca^2+^ release ↑; mitochondrial overload ↑	MCF-7 breast cancer	Mitochondrial destruction ↑; enabling FL/PA imaging	[80]

Taken together, calcium-centered nanomedicine represents the most developed branch of ion channel nanotherapy, but its mechanisms are not equally direct. TRPV1, TRPM8, TRPM2, TRPA1, TRPC6, PIEZO1 and T-type Ca^2+^ channel systems can be discussed as channel-level regulation when agonists, blockers, siRNA, channel antibodies or antagonist controls link a defined channel to Ca^2+^ influx, Ca^2+^ suppression, apoptosis, immune remodeling or reversal of resistance. Among these, TRPV1-based activation and blockade are especially mature because nanomaterials can couple channel gating with spatially confined heat, acidity, NO release, CAP delivery or membrane anchoring. RNAi-based T-type Ca^2+^ channel silencing and blocker-loaded systems also provide direct mechanistic evidence, particularly in drug-resistant or thermotolerant tumors. By contrast, calcium-supplying platforms, ROS-amplified calcium disturbance and efflux-blocking systems often produce strong antitumor effects but do not always identify one specific channel as the primary target. These designs are better interpreted as ionic-homeostasis disruption rather than direct channel modulation. Future studies should therefore quantify channel currents and Ca^2+^ flux more directly, include channel blockade or rescue controls, distinguish extracellular influx from intracellular store release, and evaluate whether calcium overload can be controlled in tumor cells without damaging normal excitable or immune tissues.

## Chloride channel dysregulation and anion imbalance in cancer nanotherapy

Chloride channels regulate V_mem_, cell volume, organelle acidification and anion transport, thereby influencing tumor proliferation, stress adaptation and therapy resistance [81]. In nanomedicine, Cl^⁻^ channel regulation is used either to block pro-survival chloride currents or to disturb compartmental chloride homeostasis and drug handling.

### TMEM16A calcium-activated chloride channels

**Fig. 5:**
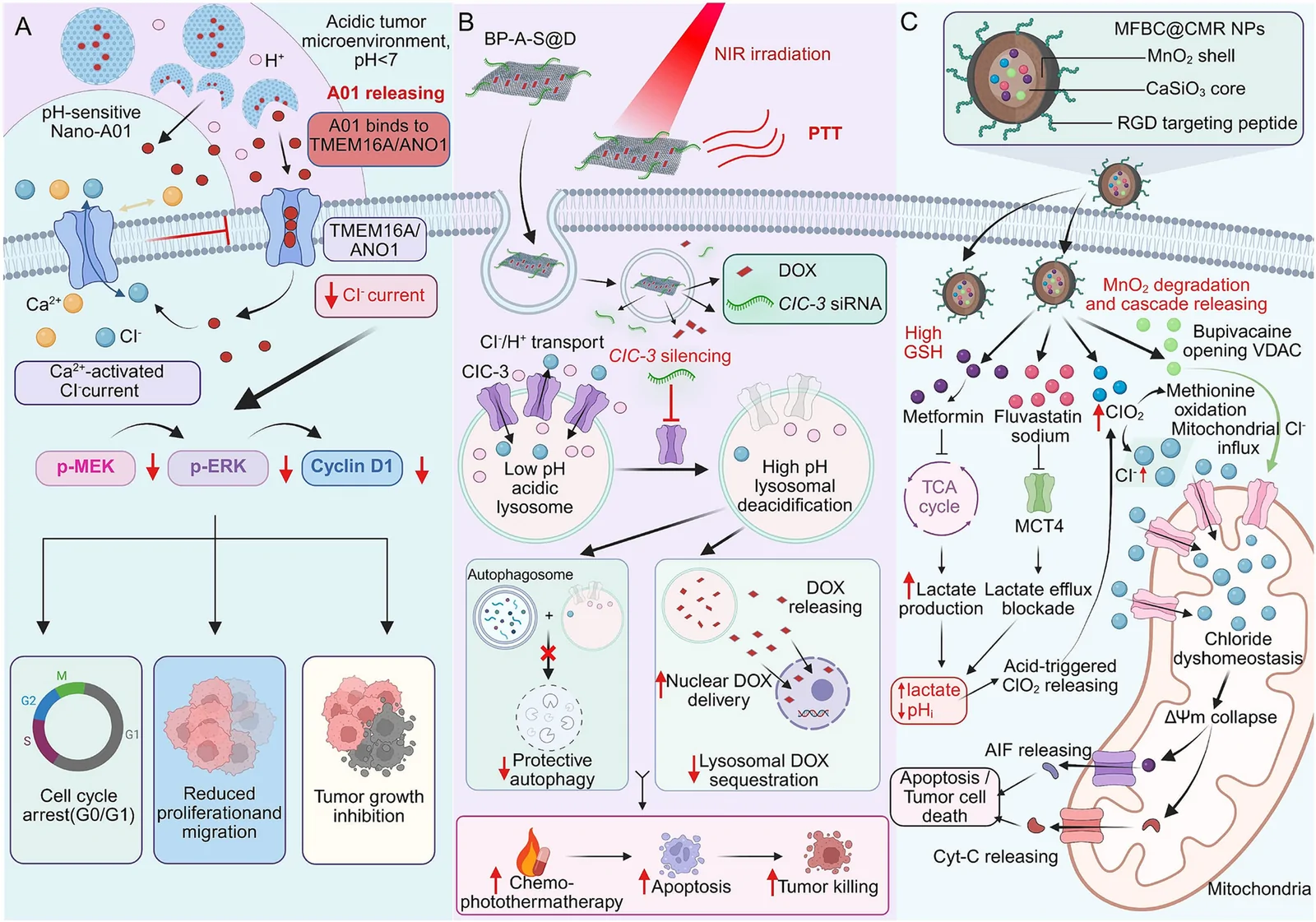
Chloride channel dysregulation and anion imbalance in cancer nanotherapy. (**A**) TMEM16A/ANO1-mediated Ca^2+^-activated Cl⁻ currents support tumor proliferation and survival signaling. pH-responsive or NIR-activated nanoplatforms deliver TMEM16A inhibitors or channel-regulating agents to suppress CaCC activity, inhibit MEK/ERK and Cyclin D1 pathways, induce cell cycle arrest and reduce tumor growth. (**B**) ClC-3 maintains lysosomal Cl^⁻^/H^+^ transport, acidic pH, protective autophagy and drug sequestration. *ClC-3* siRNA-loaded black phosphorus nanosheets disrupt lysosomal acidification, reduce autophagy, promote DOX release and enhance chemo-photothermal tumor killing. (**C**) RGD-modified MFBC@CMR nanoparticles induce mitochondrial chloride dyshomeostasis by releasing bupivacaine to open VDACs, while metformin/fluvastatin-driven acidification and MCT4 blockade trigger ClO_2_-mediated Cl^⁻^ generation. Mitochondrial Cl⁻ influx collapses ΔΨ_m_, releases Cyt-C and AIF, and induces apoptotic tumor cell death. Together, these strategies illustrate how nanomedicine reprograms chloride channels and anion homeostasis to enhance antitumor efficacy

TMEM16A/ANO1 is a Ca^2+^-activated Cl^⁻^ channel that converts intracellular Ca^2+^ signals into chloride currents and pro-tumor signaling [82]. pH-sensitive nano-assembled carriers release TMEM16A inhibitors within LUAD to suppress calcium-activated chloride channel signaling. Inhibition of overexpressed TMEM16A/calcium-activated chloride channels (CaCCs) disrupts chloride channel activity associated with tumor growth and damage resistance. Channel blockade weakens TMEM16A-dependent tumor survival pathways, producing marked antitumor activity in xenograft models without evident body-weight loss **(**Fig. 5A**)** [83]. In addition, a HA-modified carrier-free polyphenol nanonetwork (PFGH) integrates procyanidin (PC), Fe^3+^ coordination and gefitinib to co-target the TMEM16A–EGFR activation loop in breast cancer. The CD44-targeted PFGH nanosystem enables pH-responsive drug release and NIR-enhanced photothermal action, suppressing TMEM16A chloride channel currents while inhibiting EGFR signaling. Coordinated channel blockade and pathway inhibition reduce proliferative signaling, remodel apoptosis- and cancer-related transcriptional programs, and enhance tumor growth suppression in vivo [84]. PC encapsulation also enables remote TMEM16A regulation in NIR-responsive conjugated polymer nanoparticles (CPNs-PC). PC acts as a selective TMEM16A inhibitor through electrostatic interactions with key channel residues, while nanoparticle encapsulation improves its bioavailability. Under NIR irradiation, CPNs-PC suppresses TMEM16A-dependent signaling, arrests cell cycle progression and enhances tumor inhibition through targeted modulation of anion channel activity [85].

### Chloride homeostasis and resistance-associated channels

Beyond TMEM16A, chloride-associated nanomedicine turns to anion channel functions in lysosomal pH control, platinum resistance, mitochondrial chloride transport and tumor-associated channel blockade. For example, AS1411-modified black phosphorus nanosheets loaded with *ClC-3* siRNA and DOX (BP-A-S@D) silences *ClC-3* to disrupt lysosomal Cl^⁻^/H^+^ transport and arrest tumor lysosomal acidification. ClC-3 inhibition blocks the acidic lysosomal environment required for protective autophagy, sensitizing tumor cells to chemo-photothermal stress. Loss of lysosomal acidification also reduces DOX sequestration and promotes drug release from lysosomal trapping. This lysosomal ion-transport intervention weakens autophagy- and compartmentalization-driven resistance, enhancing DOX-based chemo-photothermal tumor killing **(**Fig. 5B**)** [86]. At the mitochondrial level, arginine-glycine-aspatic acid (RGD)-modified metformin/fluvastatin sodium/bupivacaine/ClO_2_-loaded CaSiO_3_@MnO_2_-RGD (MFBC@CMR) nanoparticles impose chloride dyshomeostasis by releasing bupivacaine to open voltage-dependent anion channels (VDACs) and drive mitochondrial Cl^⁻^ influx. MnO_2_ degradation in GSH-rich tumors initiates drug release, while metformin and fluvastatin acidify cancer cells by promoting lactate production and blocking monocarboxylic acid transporter 4 (MCT4)-mediated lactate efflux. Acid-triggered ClO_2_ release oxidizes methionine and generates Cl^⁻^, which enters mitochondria through opened VDACs. Chloride dyshomeostasis collapses mitochondrial V_mem_, releases Cyt-C and AIF, and induces apoptotic tumor cell death **(**Fig. 5C**)** [87]. Furthermore, chlorotoxin (CTX)- and CUR-conjugated magnetic nanochains (CTX-NCs-Cur) pair imaging-guided early-lesion targeting with matrix metallo proteinases‐2 (MMP‐2)-associated chloride channel blockade in early non-small cell lung cancer. Dextran-stabilized CTX-NCs-Cur accumulate in small lung tumors and enhance magnetic resonance and fluorescence imaging contrast. CTX-containing nanochains associate with tumor MMP‐2 complexes to block Cl^⁻^ channels, limiting cancer cell growth, while CUR increases intracellular ROS. The combined channel inhibition, oxidative stress and imaging capability enable targeted suppression and visualization of early lung tumors [88].

Taken together, chloride-focused nanomedicine is still less developed than calcium-centered strategies, but it provides a distinct route for controlling tumor survival, organelle function and therapy resistance. TMEM16A-targeted systems represent the clearest channel-directed examples, because inhibitor-loaded nanoplatforms directly suppress Ca^2+^-activated Cl^⁻^ currents and related proliferative signaling. *ClC-3* silencing also provides a defined lysosomal mechanism by linking chloride transport to acidification, autophagy and drug sequestration. In contrast, mitochondrial chloride dyshomeostasis and CTX-associated chloride channel blockade involve broader anion imbalance, oxidative stress or tumor targeting, and therefore require more direct validation of channel activity. Future studies should further distinguish chloride current inhibition from downstream anion imbalance, and combine functional Cl^⁻^ flux measurements with channel-specific blockade, knockdown or rescue experiments.

## Engineered and artificial ion-transport systems in cancer nanotherapy

Beyond endogenous channel activation, blockade and gene silencing, engineered and artificial ion-transport systems create new routes for ion flow or reshape the membrane-potential landscape. In these designs, nanomaterials deliver channel-encoding genes, artificial channel molecules or membrane-integrated bioelectronic structures, making ion flux a programmable therapeutic input.

### Gene-installed mechanosensitive and optogenetic channels

Gene-installed ion-transport systems differ from endogenous channel modulation because the therapeutic conductance is first introduced into tumor cells and then gated by an external stimulus. Cationic nanoliposome-mediated delivery of MscL I92L plasmids constructs a logic AND-gated sonogene nanosystem in tumor cells, enabling ultrasound-controlled activation of engineered mechanosensitive channels. Ultrasound opens the expressed MscL I92L channels, permitting sustained Ca^2+^ influx and triggering calcium-dependent apoptotic signaling. By requiring both channel expression and acoustic stimulation, this sonogene nanosystem confines ion channel activation to selected tumor cells and promotes controlled cancer cell apoptosis [11]. Similarly, an iron alginate (FeAlg)/polyethylenimine (PEI) nanogel system delivers MscL plasmids (FeAlg/PEI/MSCL) into tumor cells for ultrasound-triggered mechanosensitive channel activation. MscL opening induces sustained Ca^2+^ influx, selectively triggering tumor cell apoptosis. Apoptotic fragments act as tumor-associated antigens, enhancing DC maturation and activating CD8^+^ T cells up to 17.7% and 17.6%, respectively, demonstrating potent and safe antitumor immune responses [89].

Gene-installed conductance can also be controlled optically. UCNP-MOF nanohybrids introduce genetically encoded, light-controlled cation conductance by delivering plasmid DNA that installs photoactivatable channels in cancer cells. In acidic tumors, MOF degradation releases metal cations, while UCNPs convert NIR light into visible emission to open the newly expressed channels. Light-gated cation influx supplies remote-controlled ion therapy, disrupting tumor cell activity and suppressing subcutaneous and orthotopic tumor growth through optogenetically regulated channel activation [12].

### Artificial channels and bioelectronic membrane-potential modulation

Engineered ion-transport systems move beyond endogenous channel modulation by creating new ion-conducting routes or changing the V_mem_ landscape in which native channels operate. A TPP-modified block polymer carrier (MPTPP) selectively delivers the artificial potassium channel molecule 5F8 to cancer cell mitochondria. On the mitochondrial membrane, 5F8 assembles into K^+^-selective channels, driving mitochondrial K^+^ influx and disrupting ion homeostasis. The resulting mitochondrial depolarization, ATP depletion, ROS elevation and suppression of BCL-2/MCL-1 anti-apoptotic signaling induce programmed apoptosis in multidrug-resistant cancer cells [90]. Rather than introducing an artificial channel, V_mem_ modulation reshapes the bioelectric environment in which native channels operate. Carbon nanotube porins (CNTPs) act as wireless bipolar biomaterials that interface with cancer cell membranes to tune V_mem_ and ionic flux. By perturbing ion channel-linked bioelectric signaling, CNTPs combined with external voltage reshape cancer cell physiology, with invasive cells showing heightened V_mem_ responsiveness and less invasive cells exhibiting increased metabolic activity. In GBM cells, these bioelectric interventions further alter cell-cycle dynamics around 48 h, highlighting membrane-integrated nanomaterials as a strategy for regulating malignant behaviour through ion channel modulation [91].

Unlike endogenous channel-targeted platforms, engineered and artificial ion-transport systems do not rely solely on native channel expression. They introduce or construct conductance routes that can be gated by ultrasound, light, artificial channel assembly or external voltage. Their main advantage is programmability, whereas their current limitation lies in the relatively early evidence base compared with endogenous TRP or TMEM16A regulation. Future studies should define the magnitude and duration of induced ion flux, tumor selectivity of introduced channels, and safety of artificial conductance or bioelectronic membrane-potential modulation. Across the mechanisms discussed above, ionic regulation is translated into several recurrent antitumor responses, including calcium overload, mitochondrial dysfunction, membrane-potential disruption, chloride dyshomeostasis, immune activation and reversal of drug resistance (Table 2).

**Table 2:** Summary of representative nanoplatforms classified by therapeutic outcomes in ion channel-mediated cancer therapy

Therapeutic outcome	Representative nanoplatforms	Ion targets / mechanisms	Key effects	Refs
Calcium overload	CAP-loaded CaCO_3_ NPs; CSPN; CAMcaP; AuNR-DOX; I/B@MSN-T@HA	TRPV1 activation (CAP release, heat, or NO); TRPM2 activation (lysosomal Cl⁻ burst); Ca^2+^ release + efflux inhibition	Cytosolic and mitochondrial Ca^2+^ overload, Cyt-C release, caspase-3 activation, apoptosis	[13, 14, 70] [51, 76]
Mitochondrial dysfunction	K_V_1.3-targeted NIR-II NPs (PAP-1 release); Fe-PDA@CaCO_3_; GMCP; CaO_2_@PCN	Mitochondrial K⁺ channel blockade; ROS-amplified Ca^2+^ efflux suppression; SDT-induced Ca^2+^ channel dysregulation	ΔΨ_m_ collapse, ATP depletion, ROS storm, mitochondrial apoptosis	[8, 73] [77, 78]
Membrane potential collapse	Ultrasound-activated piezoelectric NPs; MPTPP@5F8 (artificial K^+^ channel); CNTPs	K_ir_3.2 upregulation; mitochondrial K^+^ influx; bioelectric interface modulation	V_mem_ disruption, cell cycle arrest, altered excitability	[30, 90, 91]
Chloride dyshomeostasis	TMEM16A inhibitors (CPNs-PC, PFGH); MFBC@CMR (VDAC opening); BP-A-S@D (*ClC-3* siRNA)	CaCC inhibition; mitochondrial Cl^⁻^ influx via VDAC; lysosomal Cl^⁻^/H^+^ transport blockade; endocytic uptake bypass	Anion imbalance, lysosomal acidification arrest, reduced autophagy, restored platinum sensitivity	[84–87]
Immune activation	LRT nanomedicines (T-type Ca^2+^ blockade); YmSNs@CRV (PIEZO1 agonist); FeAlg/PEI/MSCL (MscL); RC@NMVs (pyroptosis)	ER stress/calreticulin exposure; macrophage M1 repolarization; DAMPs release; gasdermin-mediated pyroptosis	DC maturation, CD8^+^ T cell infiltration, antitumor memory, inflammatory amplification	[56, 63, 79, 89]
Drug-resistance reversal	pMSNC/DOX/siRNA (T-type Ca^2+^); BP-A-S@D (*ClC-3* siRNA); IS-Micelles (TRPV1 blockade)	Ca^2+^ signaling knockdown; lysosomal acidification blockade; endocytic uptake; HSF1/HSP suppression	Reduced drug efflux, restored chemosensitivity, weakened thermotolerance	[59, 64, 65, 86]

## Challenges and future perspectives

Nanomaterials have expanded ion channel-based cancer therapy by delivering channel agonists, blockers, nucleic acids and artificial channel components to tumors or specific subcellular sites. These systems can convert channel regulation into Ca^2+^ overload, mitochondrial depolarization, chloride dyshomeostasis, V_mem_ disruption or immune remodeling. The key challenge is not channel modulation itself, but whether the resulting ionic stress can be controlled with sufficient selectivity, intensity and duration in heterogeneous tumor tissues.

A central limitation is that many current designs still treat ion channels as isolated targets. In cancer cells, however, ion-transport is organized as an adaptive network [92]. The same channel may produce different biological outcomes depending on tumor type, cell identity and therapeutic context. TRPV1 activation can trigger Ca^2+^-dependent apoptosis [93], whereas TRPV1 blockade may enhance mild photothermal therapy by weakening heat-shock defense [59]. Similarly, PIEZO1 activation can suppress metastatic behavior in cancer cells [54], whereas PIEZO1 activation in TAMs may remodel the immune microenvironment [55]. These examples indicate that channel function is context-dependent rather than intrinsically pro- or anti-tumor. More importantly, disrupting one channel may be insufficient if parallel TRP channels, store-operated Ca^2+^ entry machinery, Ca^2+^ pumps, K^+^ channels or Cl^⁻^ transporters restore ionic balance [94]. Future work should therefore move from single channel validation toward network-level ionic profiling, with attention to compensatory pathways activated during recovery.

Another unresolved issue is how to define the effective dose of ion channel nanotherapy. For these systems, dose should not be equated with nanomaterial amount, drug loading or external energy input. The more relevant pharmacodynamic variable is the ionic disturbance actually generated inside tumor cells. Light- or ultrasound-responsive platforms may show strong activation under controlled conditions, but the same external stimulation parameters can generate different intracellular ion fluxes across tumor regions because of tissue depth, vascular heterogeneity, stromal density, necrosis and oxygenation [95–97]. Cellular heterogeneity further complicates this problem, as cancer stem-like or drug-resistant subpopulations may possess stronger buffering and stress-adaptive capacity [98, 99]. A useful evaluation framework should therefore connect material activation, channel gating, ion flux amplitude, organelle failure and cell death at the level of tumor subregions and cell states, rather than relying mainly on average fluorescence signals, cell viability or tumor-volume inhibition.

Durability is a separate challenge. Most ion channel nanotherapies are tested as acute interventions, whereas clinical treatment would likely involve repeated dosing cycles. Surviving cells may adapt by altering channel trafficking [100], phosphorylation [101], membrane insertion [99], pump expression [102] or mitochondrial buffering [103]. Such adaptation could emerge rapidly, because many ion channel functions are regulated on minute-to-hour timescales [104]. Repeated-treatment studies should therefore include longitudinal readouts of channel expression, surface localization, pump activity, mitochondrial buffering and recovery kinetics. Without these measurements, transient ion overload may be mistaken for durable therapeutic control.

Safety also needs to be reframed. The relevant question is not only whether the nanocarrier is biocompatible, but whether tumor cells have a lower tolerance threshold for ionic disruption than normal tissues. Na^+^, K^+^, Ca^2+^ and Cl^⁻^ homeostasis is essential for cardiac conduction [105], neuronal excitability [106], epithelial transport [107], immune activation [108] and organelle function [109]. For example, *SCN5A*/Na_V_1.5 is central to cardiac conduction, and its alteration is associated with arrhythmic and cardiomyopathic disorders [110]. Therefore, the therapeutic window should be defined by differential ionic tolerance: how much Ca^2+^ overload, K^+^ redistribution, Cl^⁻^ dyshomeostasis or V_mem_ disruption tumor cells can tolerate compared with normal cells. Safety studies should incorporate ion flux amplitude, recovery capacity, electrophysiological disturbance, inflammatory response and long-term homeostatic compensation, rather than relying mainly on body weight, serum biochemistry and histology.

**Fig. 6:**
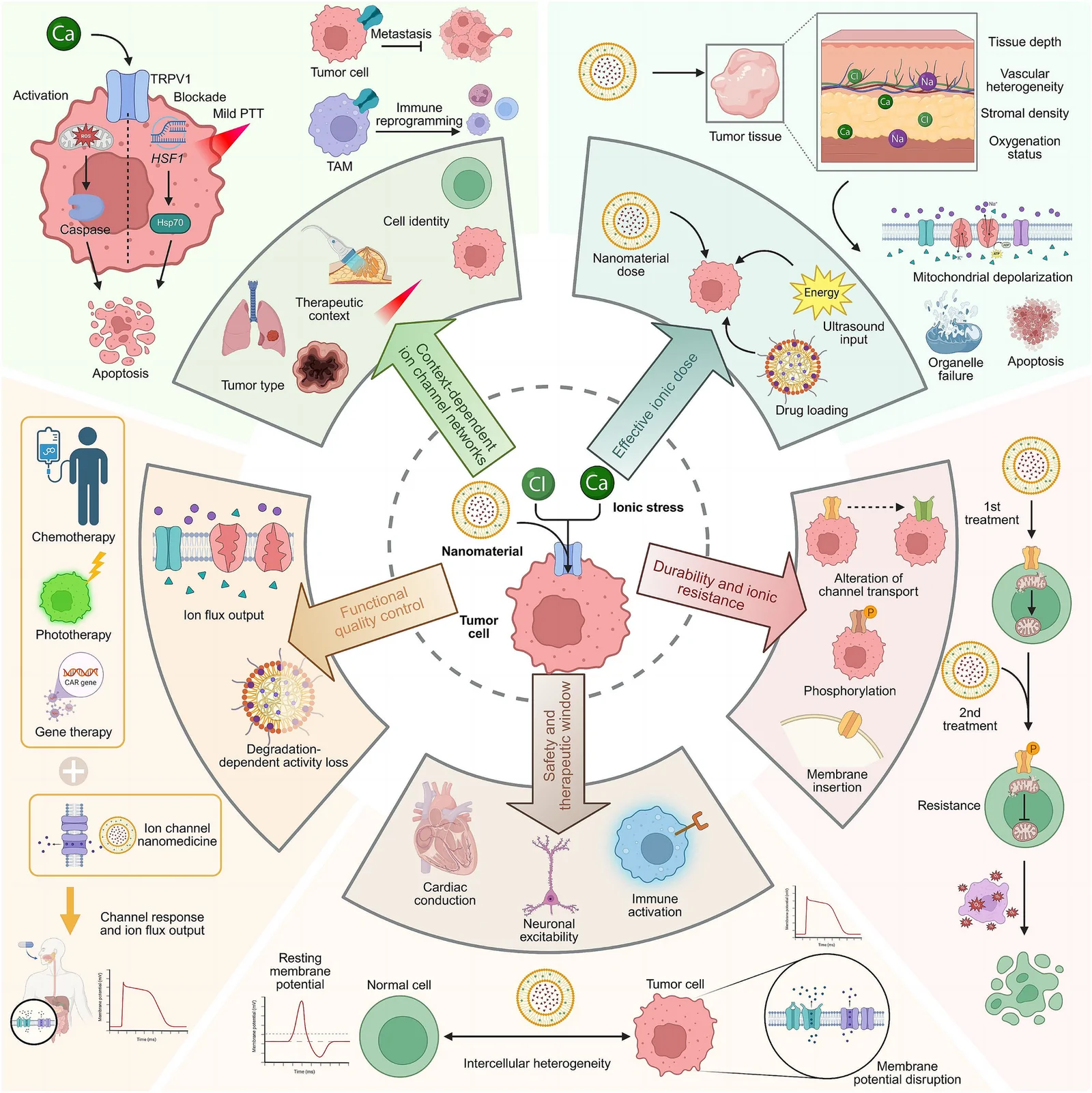
Key challenges and future directions in ion channel nanomedicine for cancer therapy. First, channel function is highly context dependent, with therapeutic outcomes shaped by tumor type, cell identity and treatment setting. Second, effective dosing should be defined by the intracellular ion flux and ionic stress generated in tumor cells, rather than only by nanomaterial amount, drug loading or external energy input; this response may vary across tumor regions because of tissue depth, vascular heterogeneity, stromal density and oxygenation. Third, repeated treatment may promote ionic resistance through adaptive changes in channel trafficking, phosphorylation, membrane insertion or related regulatory processes. Fourth, safety evaluation should define the differential ionic tolerance of tumor and normal cells, given the essential physiological roles of Na^+^, K^+^, Ca^2+^ and Cl^⁻^ homeostasis. Finally, clinical translation will require functional quality control that measures channel response, ion flux output, stimulus sensitivity and activity stability as product-level attributes

Finally, clinical translation will require ion channel modulation to be defined as a measurable product function. Conventional nanomedicine quality control can describe size, polydispersity index, charge, morphology, loading and release [111, 112], but these parameters do not prove that a formulation reproducibly generates the intended ionic event. Ion channel nanomedicine adds functional attributes such as stimulus sensitivity, channel-gating reproducibility, ion flux output, reversibility, degradation-dependent activity loss and storage stability. This becomes especially important when channel regulation is combined with chemotherapy, phototherapy, sonotherapy, gene silencing, imaging or artificial channel construction. For future development, ionic function should be treated as part of product identity. Batch-level assays should measure channel response and ion flux output, not only material composition. **(**Fig. 6**).**

## Conclusion

Ion channel nanomedicine offers a way to treat cancer by controlling ionic homeostasis rather than only delivering cytotoxic agents. Across calcium-centered homeostasis-disrupting systems and emerging K^+^, Na^+^, Cl^⁻^ and engineered ion-transport platforms, nanomaterials can localize channel regulation, activate it under external or tumor-specific cues, and couple ion flux with mitochondrial dysfunction, apoptosis, immune remodeling or other antitumor responses. This makes ion channels more than molecular targets; they become functional entry points through which material design can reshape cancer cell stress and therapeutic vulnerability. At the same time, these systems remain difficult to translate because ionic responses are highly dependent on channel subtype, tumor context, stimulation conditions and cellular buffering capacity. Future studies therefore need to define not only whether a nanoplatform inhibits tumor growth, but also whether it generates the intended ion channel event with sufficient selectivity, durability and safety. With better control over where, when and how ion flux is imposed, nanomaterial-mediated channel regulation may develop into a more precise strategy for converting cancer-associated ionic vulnerabilities into therapeutic benefit.

## Data Availability

No datasets were generated or analysed during the current study.

## Abbreviations

5-FU: 5-Fluorouracil
ADR: Adriamycin
AML: Acute myeloid leukemia
ANO1: Anoctamin-1
ATP: Adenosine triphosphate
BSA: Bovine serum albumin
CaCCs: Calcium-activated chloride channels
CaP: Calcium phosphate
CAP: Capsaicin
CDDP: Cisplatin
CGRP: Calcitonin gene-related peptide
CUR: Curcumin
CXCR4: CXC chemokine receptor 4
Cyt-C: Cytochrome C
DAMPs: Damage-associated molecular patterns
DC: Dendritic cell
DOX: Doxorubicin
EF2K: Eukaryotic elongation factor-2 kinase
ER: Endoplasmic reticulum
FL/CT: Fluorescence/computed tomography
GBM: Glioblastoma
GSH: Glutathione
HA: Hyaluronic acid
HCC: Hepatocellular carcinoma
HSF1: Heat shock transcription factor 1
HSP: Heat shock protein
ICG: Indocyanine green
IL: Interleukin
IP_3_R: Inositol 1,4,5-trisphosphate receptor
LUAD: Lung adenocarcinoma
MCT4: Monocarboxylic acid transporter 4
MDR: Multi-drug resistance
MMP-2: Matrix metalloproteinase-2
MOF: Metal–organic framework
MscL: Mechanosensitive channel of large conductance
NIR: Near-infrared
NO: Nitric oxide
NPs: Nanoparticles
PA: Photoacoustic
PAG: Photoacid generator
PC: Procyanidin
PDA: Polydopamine
PDT: Photodynamic therapy
PEG: Polyethylene glycol
PEI: Polyethylenimine
PIEZO1: Piezo type mechanosensitive ion channel component 1
PLGA: Poly(lactic-co-glycolic acid)
PTT: Photothermal therapy
RGD: Arginine-glycine-aspartic acid
ROS: Reactive oxygen species
SDT: Sonodynamic therapy
siRNA: Small interfering RNA
TAMs: Tumor-associated macrophages
TCR: T cell receptor
TGF-β: Transforming growth factor β
TMEM16A: Transmembrane member 16A
TNBC: Triple-negative breast cancer
TPP: Triphenylphosphonium
TRP: Transient receptor potential
TRPA1: Transient receptor potential ankyrin 1
TRPC6: Transient receptor potential canonical 6
TRPM: Transient receptor potential melastatin
TRPV1: Transient receptor potential vanilloid 1
UCNP: Upconversion nanoparticle
UPR: Unfolded protein response
VDACs: Voltage-dependent anion channels
Vmem: Membrane potential
